# Perinuclear Arp2/3-driven actin polymerization enables nuclear deformation to facilitate cell migration through complex environments

**DOI:** 10.1038/ncomms10997

**Published:** 2016-03-15

**Authors:** Hawa-Racine Thiam, Pablo Vargas, Nicolas Carpi, Carolina Lage Crespo, Matthew Raab, Emmanuel Terriac, Megan C. King, Jordan Jacobelli, Arthur S. Alberts, Theresia Stradal, Ana-Maria Lennon-Dumenil, Matthieu Piel

**Affiliations:** 1 Institut Curie, PSL Research University, CNRS, UMR 144, F-75005 Paris, France; 2 Institut Curie, PSL Research University, INSERM U932, F-75005 Paris, France; 3 Division of Immunology, Transplantation and Infectious Diseases, San Rafaele Scientific Institute, 20132 Milan, Italy; 4 Department of Cell Biology, Yale School of Medicine, 333 Cedar Street, New Haven, Connecticut 06520-8002, USA; 5 National Jewish Health and University of Colorado, Denver, Colorado 80206, USA; 6 Laboratory of Cell Structure and Signal Integration, Van Andel Research Institute, Grand Rapids, Michigan 49503, USA; 7 Helmholtz Centre for Infection Research, Inhoffenstrasse 7, 38124 Braunschweig, Germany

## Abstract

Cell migration has two opposite faces: although necessary for physiological processes such as immune responses, it can also have detrimental effects by enabling metastatic cells to invade new organs. *In vivo*, migration occurs in complex environments and often requires a high cellular deformability, a property limited by the cell nucleus. Here we show that dendritic cells, the sentinels of the immune system, possess a mechanism to pass through micrometric constrictions. This mechanism is based on a rapid Arp2/3-dependent actin nucleation around the nucleus that disrupts the nuclear lamina, the main structure limiting nuclear deformability. The cells' requirement for Arp2/3 to pass through constrictions can be relieved when nuclear stiffness is decreased by suppressing lamin A/C expression. We propose a new role for Arp2/3 in three-dimensional cell migration, allowing fast-moving cells such as leukocytes to rapidly and efficiently migrate through narrow gaps, a process probably important for their function.

## Abstract (web summary)

Cell migration through micrometric constraints is limited by low deformability of the nucleus. Here the authors show that in dendritic cells a perinuclear actin network nucleated by Arp2/3 increases nuclear deformation and allows the cells to pass through narrow constrictions, likely by rupturing the nuclear lamina.

## Graphical abstract

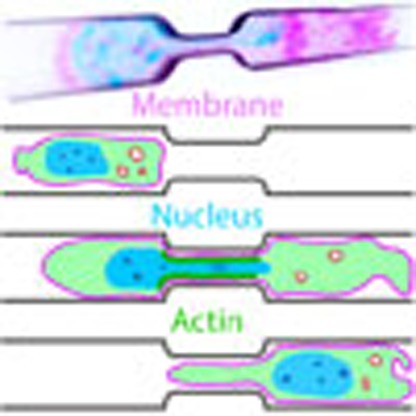

The actomyosin machinery required for cell locomotion has been described in great details^1^. It is mainly based on actin treadmilling and actomyosin contraction^2^,^3^. The net rearward movement of actin filaments is transferred to the migration substratum via specific adhesion proteins (for example, integrin receptors^4^,^5^), leading to forward cell locomotion^1^,^6^. This well-defined set of physico-chemical processes, initially described based on cultured cells moving on flat glass surfaces, seems to be universal among migrating cells. Nevertheless, recent studies performed in more complex environments have proposed alternative scenarios and additional requirements for cell locomotion, suggesting that the precise function of some proteins might depend on the environment of migrating cells. The confining nature of micro-channels, gels of the extracellular matrix or tissues can relieve the need for specific integrin-based adhesion in immune and cancer cells^7^,^8^,^9^,^10^. The complexity of three-dimensional (3D) gels also requires cells to restrict the number of their protrusions, to avoid simultaneously engaging multiple paths. This constraint makes polarity proteins such as Cdc42 essential for 3D locomotion, whereas on two-dimensional substrates it is required only for directionality^11^.

In addition, the porous nature of gels and tissues places a strong requirement on cell deformability/compliance^12^. Owing to the dynamic nature of the actin and microtubule cytoskeletons, the high compliance of the plasma membrane and of the majority of membranous internal organelles, most cytoplasmic elements do not limit cell deformation through the micrometric pores found in tissues^13^, at least on the time scale of cell locomotion. Cytoplasmic intermediate filaments can limit cell deformation^14^,^15^; however, several studies have shown that the element that usually limits deformation through narrow pores is the cell nucleus^16^,^17^. Indeed, the nucleus has a specific set of intermediate filaments that form a rigid shell underneath the inner nuclear membrane; these filaments are encoded by the genes for lamin A/C and B (*LMNA* and *LMNB1/2*).

To overcome this limitation, some migrating cells express a low level of lamin gene products, whereas others secrete proteases to enlarge pores in the extracellular matrix^18^. Matrix degradation is important for the migration of metastatic cells, but does not seem to be required for immune cell migration^17^,^19^. Although the degree of lamin A/C expression correlates with the capacity of cells to pass through small pores in a protease-independent manner^16^,^17^,^20^, low levels of lamin A/C also correlate with lower cell viability^16^. Immune cells such as neutrophils do not express lamin A/C and have a short lifespan (dying at the site of infection); however, dendritic cells (DCs), which patrol peripheral tissues and present antigens to T cells in lymph nodes, need to combine a high migration capacity and long-term survival, a combination which might also be essential for the metastatic capability of some cancer cells^16^.

We here aimed at studying the mechanism used by DCs to deform and migrate through narrow pores. Our study reveals that, as already reported for various other cell lines^16^,^17^,^21^,^22^ the nucleus limits confined 3D migration in DCs. We show that DCs overcome this physical limitation by generating a dense and dynamic perinuclear actin network nucleated by Arp2/3 downstream of Wave2. This Arp2/3-nucleated perinuclear actin network allows nuclear deformation and the subsequent cell passage through constrictions, probably by rupturing the intranuclear lamina shell.

## Results

### The nucleus limits DC migration through micrometric pores

Mouse bone-marrow-derived DCs express higher levels of lamin A/C proteins compared with neutrophils^17^ or B lymphocytes (Supplementary Fig. 1A,B). However, they have a lower expression level of lamin A/C proteins than mouse embryonic fibroblasts (Supplementary Fig. 1A,B) or cultured cells used as model systems for tumour invasion (fibrosarcoma cells HT1080 and breast adenocarcinoma MDA-MB231; Supplementary Fig. 1A,B), which are slow-migrating cells that require matrix proteases for migration in dense 3D collagen gels^17^. DCs display an intermediate level of lamin A/C expression, consistent with a double requirement for a longer lifespan and a high migratory capacity independent of proteases^19^. This raises the question of how such lamin A/C-expressing cells deform their nucleus through small pores. Actomyosin contraction was proposed to play such a role, generating a pressure at the cell back that pushes the nucleus forward^23^,^24^. Myosin II is also required for cell locomotion per se^17^,^25^,^26^, and for cell polarity and retraction of lateral protrusions^27^, making it difficult to clearly identify a specific role for it in nuclear deformation.

**Figure 1:**
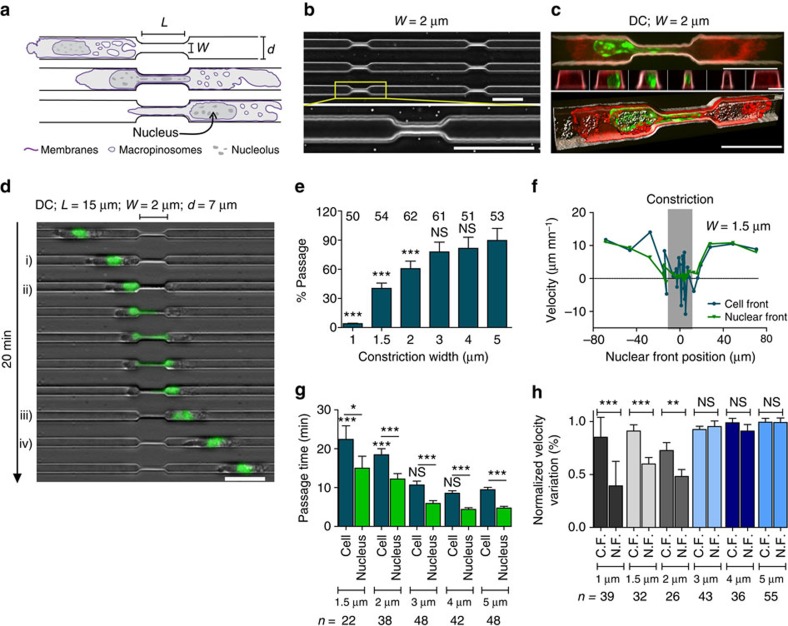
The nucleus imposes a physical limitation to DC migration through micrometric pores. (**a**) Schematic representation of the experimental setup. (**b**) Top: representative image of a field of channels with constrictions. Bottom: zoom on a single constriction. Scale bar, 30 μm. (**c**) Top: representative image of an Iaβ-GFP (red)-expressing DC stained with Hoechst (DNA, green), passing through a 2-μm constriction coated with pLL-PEG-Rhodamin (grey levels). Scale bar, 15 μm. Middle: views of the channel cross-section before, along and after the constriction. Scale bar, 4 μm. Bottom: perspective view (45°) of a 3D iso-surface reconstruction. Scale bar, 15 μm. (**d**) Representative sequential images of a DC stained with Hoechst (green) migrating through a constriction. (i) Cell entry, (ii) nuclear entry, (iii) nuclear exit and (iv) cell exit. Scale bar, 30 μm. Time in minutes: seconds. (**e**) Percentage of passage through 20-μm-long constrictions. Numbers above bars represent the number of cells scored. (**f**) Representative cell (blue) and nuclear (green) front instantaneous velocity as a function of the nuclear front position in 1.5 μm constrictions.(**g**) Cell (blue) and nuclear (green) passage time in 20-μm-long constrictions. (**e**,**g**) Unless when indicated by a spanner, statistical test was against the value for 5-μm-wide constrictions. (**h**) Cell and nuclear front velocities inside constrictions, normalized by the cell centre of mass velocity before the constriction. C.F., cell front; N.F., nuclear front. Error bars represent the s.e.m. (**g**,**h**) *n* represents the number of cells. ***P-value<0.0001, **P-value<0.001 and *P-value<0.01; NS,=nonsignificant. Statistical test: Fisher's test for **e**, Mann–Whitney test for **g** and **h**. Error bars are s.e.m. (**a**,**d**) *L* and *W* for constriction length and width, respectively; *d* for channel width; *N*≥2.

To ask how DCs deform their nucleus, we studied their migration through microfabricated constrictions^28^ of well-defined sizes coated with Fibronectin (Fig. 1a,b and Supplementary Figs 1C,D and 2A,B; for ranges of physiological pore sizes see refs 13, 17). DCs migrated spontaneously and at high speed (mean velocity of 4.48±3.93 μm min^−1^; mean±s.d.; *n*>100, *N*=2) in 7-μm-wide channels devoid of constrictions (see Supplementary Table 1 for all channels and constriction dimensions). Constrictions ≥1.5 μm in width induced a strong nuclear deformation but did not prevent cell passage (Fig. 1c–e, Supplementary Figs 1E–G and 2C,D, and Supplementary Movie 1; see also Supplementary Table 2 for exact mean±s.e.m. and *P*-value of all quantifications).

Time-lapse recording of migrating cells identified four phases of transmigration (Fig. 1d) as follows: (i) cell front entry into the constriction, which did not induce any slowdown; (ii) nuclear engagement and deformation, which led to a strong slowdown in constrictions <3 μm in width (Fig. 1f–h); (iii) nuclear exit; and (iv) cell exit. Varying the length of the constriction, from 15 μm to either 20 μm or 5 μm, did not significantly affect cell passage (compare Fig. 1 and Supplementary Fig. 1 with Supplementary Fig. 2). However, there was a clear effect of the constriction width (Fig. 1e–h) as previously reported in gels^17^. At 1 μm width, the cell front passed but only a small fraction of the nucleus was engaged in the constriction (Supplementary Fig. 1E and Supplementary Movie 1), leading to a very low (4%) rate of cell passage. Increasing the constriction width to 1.5 μm allowed 40% of cells to pass (Fig. 1e, Supplementary Fig. 1F and Supplementary Movie 1). The passage rate reached a maximum at ≥3 μm (78%; Fig. 1e and Supplementary Fig. 2D).

Quantifications (Fig. 1e–g and Supplementary Fig. 2D,E) showed that for intermediate sizes of 1.5 and 2 μm, while a majority of cells could pass through the constriction, nuclear passage induced a significant reduction of cell velocity (41% in 1.5 μm constrictions). Despite that delay, DCs passed through such small constrictions in only ∼20 min, while metastatic cells or fibroblasts have been reported to take several hours to pass through similar constrictions (mouse embryonic fibroblasts^21^ and MDA-MB-231 (ref. 22)). This suggests that fast immune cells might have a specific mechanism for deforming their nucleus in a shorter time. Importantly, for all sizes, non-passing DCs engaged in a constriction spent at least as much time in the constriction as passing cells (Supplementary Figs 1H and 2F), showing that non-passage was not due to an early change of direction of the cell induced by the constriction. Thus, DCs, despite their ability to express lamin A/C, pass constrictions down to ∼1.5 μm in width at high velocity. However, below 3 μm the DC nucleus is limiting their passage and speed.

### Arp2/3 is required for DC passage through micrometric pores

**Figure 2:**
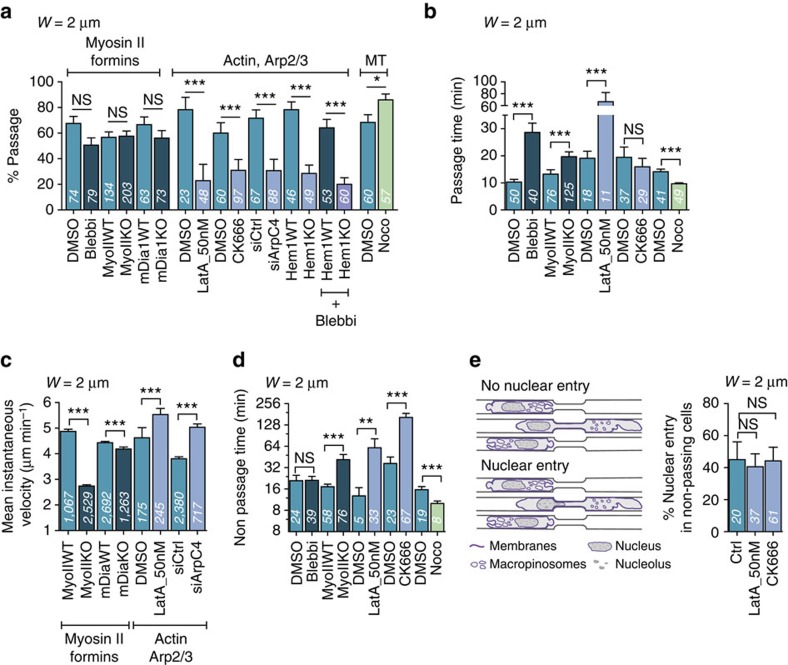
Arp2/3-based actin polymerization is required for DC passage through constrictions. (**a**) Percentage of passage and (**b**) passage time in 2-μm-wide constrictions. Blebbi, blebbistatin; LatA, latrunculin A; MT, microtubules; Noco, nocodazole. (**c**) Mean instantaneous speed in straight 7-μm-wide channels. (**d**) Non-passage time in 2-μm-wide constrictions. (**e**) Left: schematic of the nuclear entry events. Right: percentage of nuclear entry in non-passing cells. (**a**,**b**,**d**,**e**) Numbers in bars represent the number of scored cells. (**c**) Numbers in bars represent the number of cell tracks for each condition. ***P-value<0.0001, **P-value<0.001 and *P-value<0.01; NS, nonsignificant. Statistical test: Fisher's test for **a** and **e**, Mann–Whitney test for **b**,**c** and **d**. Error bars are s.e.m. *N*≥3.

To identify cytoskeletal effectors essential for nuclear passage, we performed a candidate screen using 2-μm-wide constrictions (2 × 3.5 μm^2^). Disrupting microtubules with nocodazole (Supplementary Fig. 3A) increased cell passage rate (Fig. 2a) and reduced passage time (Fig. 2b). Depletion of mDia1, the most expressed formin in DCs (Immunological Genome project http://www.immgen.org), which has been implicated in DC migration^29^, only slightly reduced immature DC speed in channels without constrictions (Fig. 2c, see also^30^) but had no effect on the rate of passage (Fig. 2a). Surprisingly, myosin II inhibition or depletion of myosin IIA (the only isoform expressed in DCs, Immunological Genome project and Supplementary Fig. 4A) had no effect on the rate of passage through constrictions (Fig. 2a and Supplementary Fig. 4B,C), even though, as expected, it had a strong effect on cell shape (Supplementary Fig. 4A,B), cell speed (Fig. 2c) and thus passage time (Fig. 2b and Supplementary Fig. 4D). We conclude that myosin II plays a role in increasing cell speed but is not required in DCs for nuclear deformation through small constrictions.

High doses of latrunculin A (250 nM), which depolymerizes actin filaments (Supplementary Fig. 3B), completely stopped cell migration (Supplementary Movie 2), ruling out an osmotically driven migration mechanism^31^. At low doses (50 nM), latrunculin A did not induce obvious changes in the overall actin cytoskeleton (Supplementary Fig. 3C and Supplementary Movie 2) neither slowed down cell movement in channels (Fig. 2c), but it prevented nuclear passage through constrictions (Fig. 2a). A similar result was obtained when the actin nucleator Arp2/3 was inhibited by CK666 (ref. 32), depleted by small interfering RNA (siRNA) (Supplementary Fig. 5A) or when the haematopoietic specific Wave2 subunit Hem1 was depleted^33^ (Fig. 2a–c). The reduction in passage rate through constrictions was not due to an increase in changes of direction, as treated/depleted cells spent at least the same amount of time in constrictions as control/wild-type (WT) cells did (Fig. 2d), and they initiated nuclear entry into constrictions to the same extent as non-passing control/WT cells (Fig. 2e). Arp2/3 inhibition reduced the rate of passage only for constrictions <3 μm (Supplementary Fig. 5B,C), a result consistent with a specific function of Arp2/3 in nuclear passage. Overall, this screen revealed that Wave2/Arp2/3-based actin nucleation is specifically needed in DCs for nuclear passage through small constrictions.

### Actin accumulates in constriction during nuclear passage

**Figure 3:**
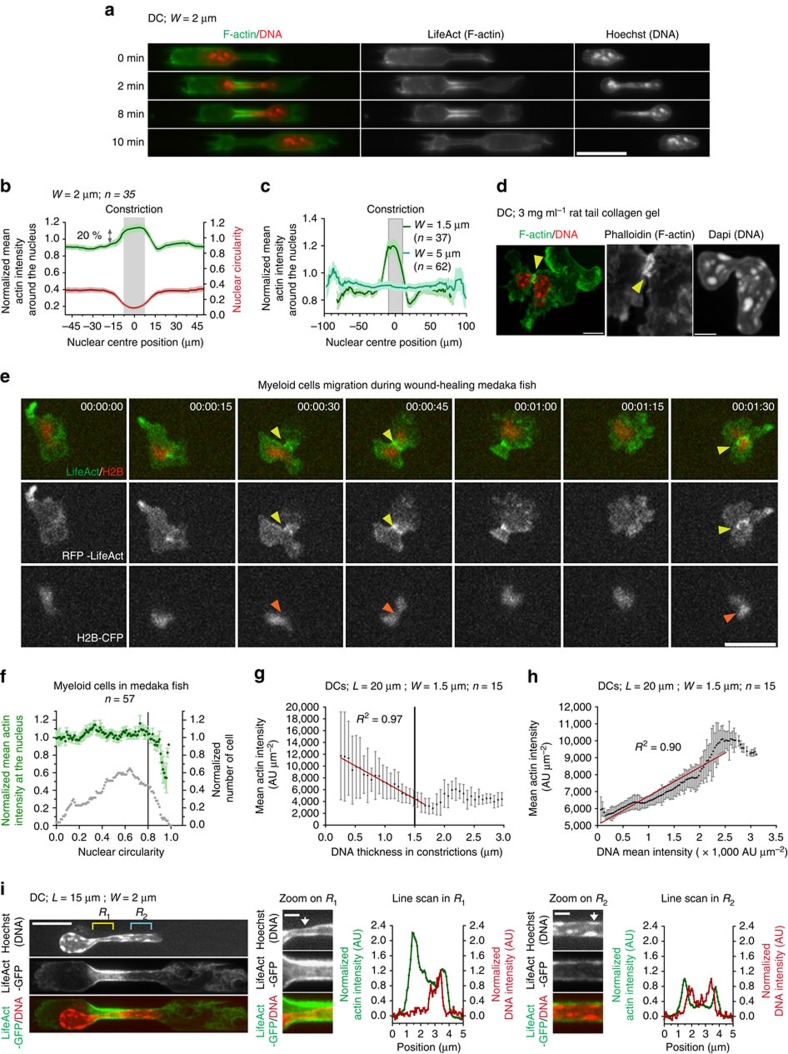
A dynamic actin meshwork forms around the nucleus and deform this organelle during its passage through narrow constrictions. (**a**) Sequential images of a representative LifeAct-GFP (green) expressing DC stained with Hoechst (red) passing a 2-μm-wide constriction. Scale bar, 20 μm. (**b**) Normalized mean actin intensity around the nucleus (green) and nuclear circularity (red) of cells crossing 2-μm-wide constrictions as a function of the nuclear centre of mass position relative to the centre of the constriction. (**c**) Normalized mean actin intensity around the nucleus in 1.5 μm (dark green) and 5 μm (light green) wide constrictions as a function of the nuclear centre of mass position relative to the centre of the constriction. (**d**) Immunostaining of a DC migrating in a collagen gel; yellow arrows show actin accumulation at the site of nuclear deformation. Scale bar, 10 μm (left); 5 μm (right). (**e**) RFP-LifeAct (green)- and H2B-CFP (red)-expressing myeloid cells migrating in mekada fish during *in vivo* wound healing. Yellow arrows indicate actin accumulation around the deformed nucleus (orange arrows, representative of 53 of the 66 observed cells). Time frames in hours: minutes: seconds. Scale bar, 15 μm. (**f**) Normalized mean actin intensity at the nuclear region in myeloid cells migrating in mekada fish as a function of nuclear circularity. Error bars are s.e.m. (**g**) Mean actin intensity as a function of DNA thickness at a position X in 1.5-μm-wide constrictions. Red line indicates the linear regression of the data for DNA tickness smaller than 1.5 μm (the constriction width). Error bars are s.d. (**h**) Mean actin intensity as a function of mean DNA intensity at a position X in 1.5-μm-wide constrictions. Red line indicates the linear regression of the data for DNA mean intensity smaller than 2,500 AU μm^−2^. Error bars are s.e.m. (**i**) Representative spinning disc images of LifeAct-GFP-expressing (actin, green) DC stained with Hoechst (DNA, red) in 2-μm-wide constrictions. Scale bar, 10 μm. White arrow indicates the position of the line scan. Scale bar, 2 μm. *L*, constriction length; *n*, number of cells; *W*, constriction width. *N*≥2.

The requirement for Arp2/3 prompted us to look in more detail at the localization of actin filaments in cells passing through constrictions. DCs derived from LifeAct-GFP transgenic mice^34^ showed a very strong enrichment of actin filaments around the nucleus inside small constrictions (Fig. 3a, Supplementary Fig. 6B and Supplementary Movie 3). This was confirmed with phalloidin staining of fixed cells (Supplementary Fig. 6A) and was quantified in live cells, showing that it was temporally restricted to the time during which the nucleus was deformed in the constriction and spatially limited to the constriction (Fig. 3b,c, Supplementary Fig. 6C and Supplementary Movies 3 and 4). This enrichment of actin was specific to small constrictions (Supplementary Fig. 6 C–N and Supplementary Movie 4) that required Arp2/3 for efficient passage (Fig. 2a and Supplementary Fig. 5B, C). This enrichment was distinct and stronger than the permanent enrichment of actin at the cell back and the transient enrichments observed at the cell front (yellow and pink arrow heads in Supplementary Fig. 6B,E, respectively). Actin accumulation around the nucleus was also observed in non-adhesive (pLL-g-PEG coated) channels (Supplementary Fig. 7A,B and Supplementary Movie 5) ruling out the involvement of adhesion complexes. Imaging of actin filaments in DCs migrating in dense, 3 mg mL^−1^ rat tail collagen gels^17^ (Fig. 3d) or in mouse ear explants (Supplementary Movie 6), and also in myeloid cells migrating in live Medaka fish, showed that the degree of nuclear deformation correlated with an accumulation of actin around the nucleus (Fig. 3e,f and Supplementary Movie 7). This suggests that a specific set of actin filaments is assembled around the nucleus of a DC when it is strongly confined *in vitro* and *in vivo*. We propose to name this network confinement induced actin network (CiAN).

### CiAN induces local nuclear deformation

To assess where forces were exerted during nuclear passage, we recorded cells passing through a constriction by reflection interference contrast microscopy. We found that cells were in close contact with the channel walls only at the rear and inside the constriction, but that the front part of the cell, which passed the constriction before the nucleus, had almost no contact with the bottom wall of the channel (Supplementary Fig. 7C and Supplementary Movie 8). This result, together with the absence of requirement for adhesion, suggests that forces exerted on the nucleus could not be pulling forces from the front protrusion, contrary to what was suggested by experiments performed on slow migrating mesenchymal cells^35^. They could instead be either pushing forces from the back or lateral forces in the constriction. To test this hypothesis, we measured, all along constrictions, both the local width and the local density of the chromatin staining, as a function of the density of the local LifeAct-GFP staining. This showed that a local higher density of actin filaments correlated with a locally thinner and denser nucleus (smaller than the actual constriction width) (Fig. 3g–i). Overall, these data suggest that actin filaments accumulated around the nucleus in the constriction exert a lateral pushing force, increasing its deformation.

### CiAN facilitates nuclear passage

**Figure 4:**
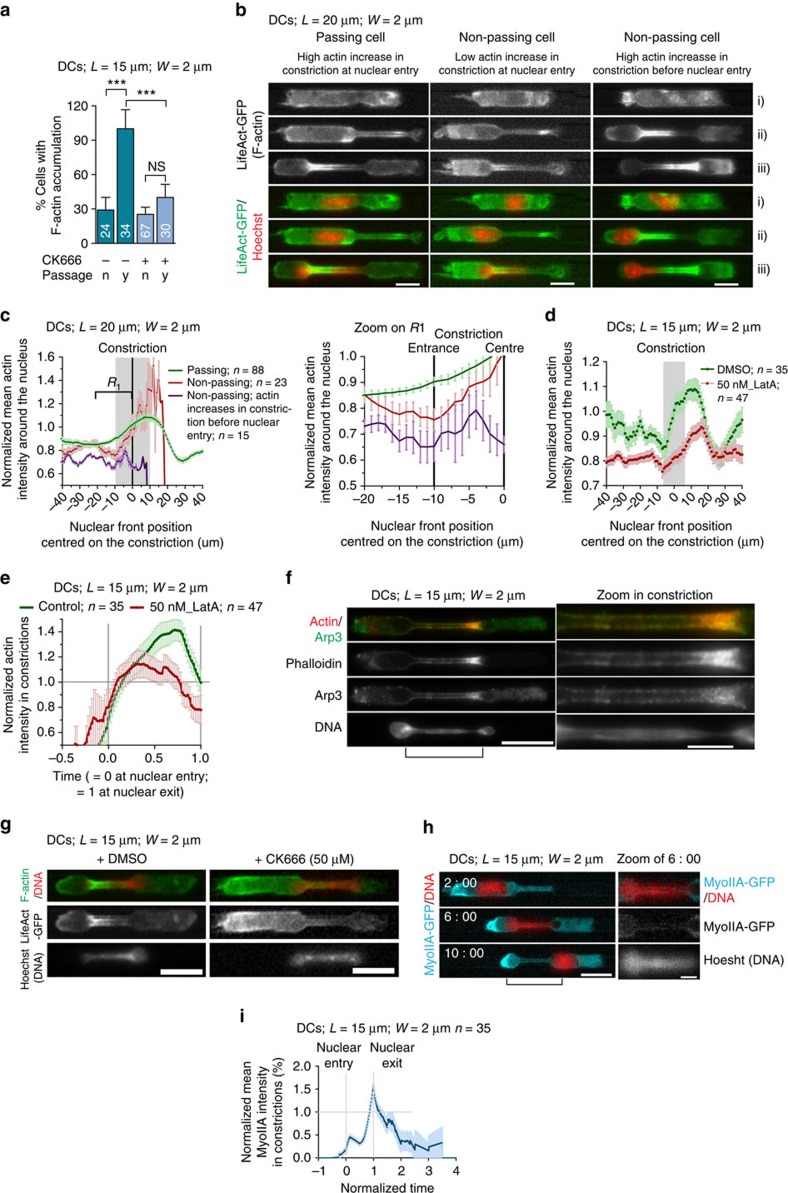
The dynamic perinuclear actin network is nucleated by Arp2/3. (**a**) Percentage of cells with F-actin accumulation in 2-μm-wide constrictions after nuclear entry. *n*, no; *y*, yes. Numbers represent the number of cells scored. ***P<0.0001 by Fisher's test. (**b**) Representative images of LifeAct-GFP (green)-expressing DCs stained with Hoechst (red) passing or failing to pass a 2-μm-wide constriction. (i) Time point before cell and nuclear entry in constriction; (ii) time point after cell but before nuclear entry in constriction; (iii) time point after cell and nuclear entry in constriction. Scale bar, 10 μm. (**c**) Left graph: normalized mean actin intensity in DCs that succeed (green) or fail (red and purple) to pass 2-μm-wide constrictions as a function of nuclear front position centred on the constriction. Grey bar represents the constriction. Right graph: zoom on constriction entry (region R1) of the left graph. (**d**) Normalized mean actin intensity in DMSO-treated (green) and 50 nM of Latrunculin A-treated (red) DCs as a function of nuclear front position centred on the constriction. Grey bar represents the constriction. (**e**) Normalized mean actin intensity in constriction measured in DMSO-treated (green) and 50 nM of Latrunculin A-treated DCs as a function of the nuclear entry and exit time. (**c**,**d**,**e**) Dark and light colours respectively represent the mean and the s.d. (**f**) Representative image of a DC immunostained for Arp2/3 and stained for actin filaments in a 2-μm-wide constriction. Scale bar, 15 μm (left); 5 μm (right). The pinched DNA at the actin accumulation site is noteworthy. (**g**) LifeAct-GFP (green)-expressing DCs stained with Hoechst (red) in 2-μm-wide constrictions. Left: control cell; right: after Arp2/3 inhibition. Scale bar, 15 μm. (**h**) Sequential images of a representative myosin IIA-GFP (cyan)-expressing DC stained with Hoechst (red). Scale bar, 15 μm (left); 5 μm (right). (**i**) Normalized mean myosin IIA-GFP (blue) intensity in 2-μm-wide constrictions. *L*, constriction length; *n*, number of cells; *W*, constriction width. *N*≥2. All error bars are s.e.m.

This result prompted us to record the perinuclear actin network in non-passing cells. More than 90% of control cells passing through constrictions displayed an actin accumulation around the nucleus versus <30% of non-passing cells that engaged their nucleus in the constriction (Fig. 4a–c). Interestingly, 58% of cells that did not form a perinuclear actin network at the moment of nuclear entry displayed a higher actin density in constriction before nuclear entry (Fig. 4b). This subpopulation of cells nucleating actin in constrictions before nuclear entry was never observed in the 88 analysed passing cells. The remaining 42% did not show an increase in actin density in constriction neither prior nor when the nucleus was inside the constriction. When measuring the density of actin around the nucleus over time, we found that non-passing cells had in average a lower density of perinuclear actin network than passing cells (Fig. 4c; it is noteworthy that the amount of perinuclear actin in non-passing cells was increasing for nuclei, which reached further inside the constriction). These results suggest that cells that did not pass constrictions either did not assemble actin around their nucleus but in front of it, or assembled actin at a lower rate and in average up to a lower level than passing cells.

We then recorded actin density around the nucleus in cells treated with 50 nM latrunculin A (Supplementary Fig. 8 and Supplementary Movie 9). We found that the density of perinuclear actin increased at a much lower rate and up to a lower maximal density than in control cells (Fig. 4d; it is noteworthy that even for treated cells the amount of actin increased for nuclei that reached further in the constriction), leading to a lower average actin accumulation inside constrictions (Fig. 4e). We also observed two patterns of actin accumulation around or in front of the nucleus in latrunculin A-treated cells that failed to pass constrictions (Supplementary Fig. 8B,C). Together, these results suggest that the amount of actin accumulation around the nucleus inside constrictions is strongly correlated with the fate of the cell (passing or not). This is consistent with a role for perinuclear actin in helping nuclear passage through the constriction.

### Arp2/3 mediates CiAN assembly while myosin II does not

As Arp2/3 complex depletion/inhibition had a strong effect on nuclear passage, we tested whether it was responsible for the assembly of the perinuclear actin network. We immunostained Arp3 in cells fixed inside constrictions, after removal of the confining polydimethylsiloxane (PDMS) layer, to allow a direct access for the antibody to the cells (see Methods). This revealed a clear enrichment of the Arp2/3 complex around the nucleus, co-localized with the enrichment in actin filaments stained by phalloidin (Fig. 4f and Supplementary Fig. 9A; *n*=10). Of cells that passed through constrictions after Arp2/3 inhibition, only a minor fraction still displayed an actin accumulation around the nucleus (Fig. 4a,g, Supplementary Fig. 9B,C and Supplementary Movie 10). These results suggest that Arp2/3 might be responsible for the increase in actin density around the nucleus of cells passing constrictions.

Consistent with a requirement for myosin II for cell speed and not for nuclear passage, myosin IIA-GFP, imaged in DCs derived from myosin IIA-GFP transgenic mice^36^, was strongly enriched at the cell back when cells were passing constrictions and did not show any enrichment around the nucleus inside the constriction (Fig. 4h,i, Supplementary Fig. 10A and Supplementary Movie 11). Moreover, inhibition of myosin II with blebbistatin did not prevent actin accumulation around the nucleus in constrictions (Supplementary Fig. 10B; representative of 19 over 21 observed cells), whereas it completely inhibited myosin II accumulation at the cell rear (Supplementary Fig. 10C; *n*=30; *N*=2). These results suggest that the actin accumulation observed around the nucleus in constrictions relies on Arp2/3 activity and corresponds to accumulation of newly polymerized actin filaments rather than to a contractile acto-myosin ring.

Thus, in DCs, myosin II motor activity, but not Arp2/3-based actin nucleation, is required for fast locomotion^24^,^30^, probably by increasing the rear contractility of the cell, whereas Arp2/3 actin nucleation around the nucleus, but not myosin II motor activity, allows efficient passage through small constrictions, probably by generating a lateral pressure on the nucleus.

### Nuclear passage leads to transient nuclear lamina rupture

**Figure 5:**
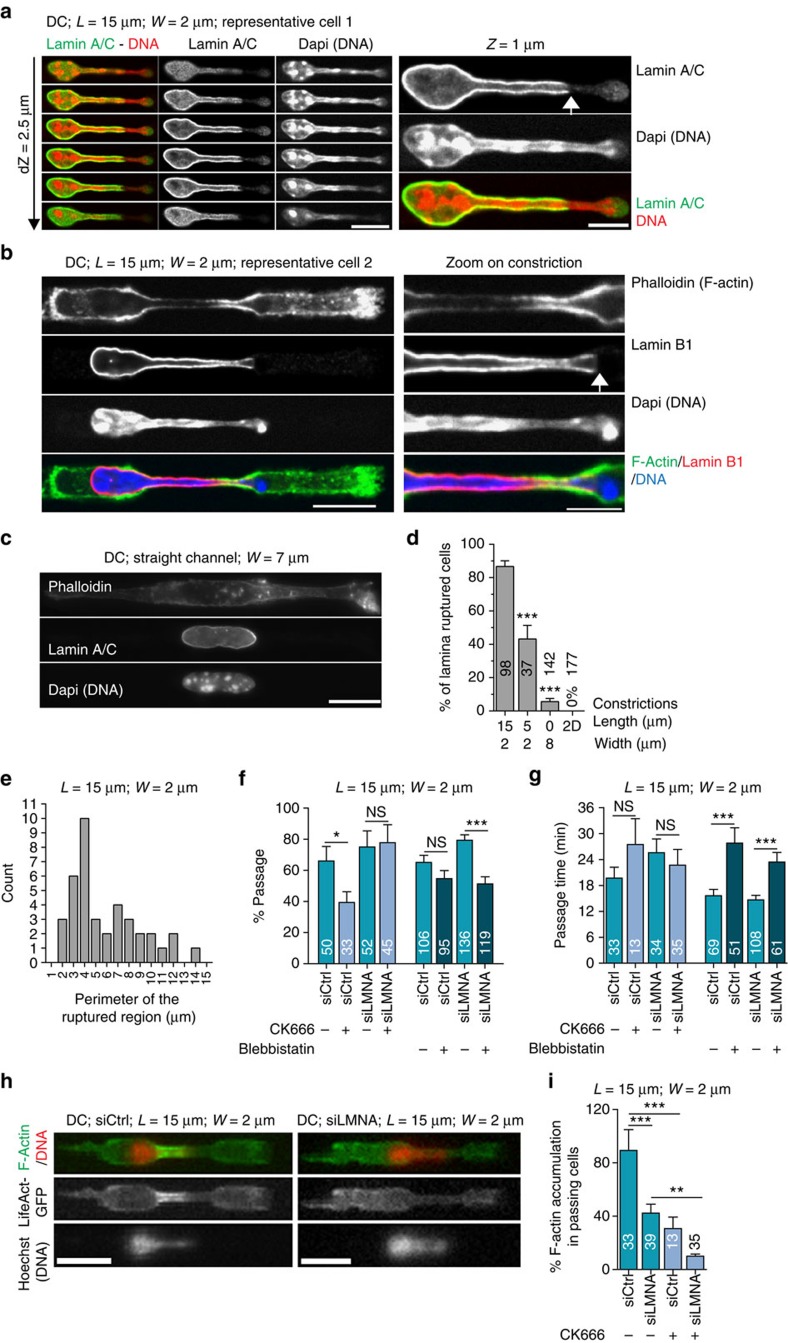
Arp2/3-nucleated perinuclear actin facilitates nuclear squeezing through constrictions by weakening the lamin A/C network. Representative images of immunostained DCs (**a**), (**b**) in 2-μm-wide constrictions and (**c**) in 8-μm-wide channels. White arrows indicate region of ruptured lamin A/C or Lamin B1 network. Scale bars10 μm (**a**,**b** (left panel) and **b**); 5 μm (**a**,**b** (right panel)). (**d**) Percentage of DCs showing a ruptured lamina network in 2-μm-wide and 15- or 5-μm-long constrictions, in straight 8-μm-wide channels and on two-dimensional substrates. (**e**) Histogram of the perimeter of the ruptured lamina region in 2-μm-wide and 15-μm-long constrictions. (**f**) Percentage of passage and (**g**) passage time in 2-μm-wide constrictions of DCs treated as indicated. (**h**) LifeAct-GFP (green)-expressing DCs stained with Hoechst (red). Left: control siRNA; right: laminA/C siRNA. Scale bar, 15 μm. (**i**) Percentage of passing cells with F-actin accumulation in 2-μm-wide constrictions. siCtrl, control siRNA; siLMNA, Lamin A/C siRNA. (**d**,**f**,**g**,**i**) Numbers represent the number of cells observed. Error bars are s.e.m. ***P-value<0.0001, **P-value<0.001 and *P-value<0.01. NS, nonsignificant. Statistical test: Fisher's test for **c** and **f**, Mann–Whitney test for **d**. *L*, constriction length; *W*, constriction width.. *N*≥3.

To further understand how actin nucleation around the nucleus helps nuclear deformation, we stained lamin A/C and lamin B in fixed cells inside constrictions. The perinuclear lamina staining was interrupted or fragmented (Fig. 5a,b and Supplementary Fig. 11A–C) in 87% of the 98 observed cells with nuclei engaged in 2-μm-wide and 15-μm-long constrictions, whereas such staining was observed in only 7% of the 142 cells observed in straight, 8-μm-wide channels and was never observed in the 177 cells observed outside of channels (Fig. 5c,d). Only 43% of the cells engaged in 2-μm-wide but 5-μm-long constrictions showed a ruptured lamina network (Fig. 5d and Supplementary Fig. 11D), suggesting that increased ruptures correlate with increased global nuclear deformation (probably due to the increase in total surface area) and not only with the local minimal width of the constriction.

In 97% of cells, the ruptured region was located at the nuclear tip (Fig. 5a–e and Supplementary Fig. 10), with part of the chromatin extruding out of the nuclear lamina staining, consistent with what was observed when cells were externally compressed^37^. These data suggest that the accumulation of actin filaments might be required to deform the lamina network, possibly by exerting pressure on it^38^, leading to its rupture at the site of highest curvature at the nuclear tip. Overall, these results suggest that on nuclear deformation, the nuclear lamina ruptures, which might facilitate the passage of the nucleus.

To directly test this hypothesis, we depleted lamin A/C in DCs (Supplementary Fig. 12) and inhibited Arp2/3. In contrast to control cells, inhibition of Arp2/3 did not prevent passage through small constrictions in lamin A/C-depleted cells (Fig. 5f). Depletion of lamin A/C did not affect the time required for passage through constrictions (Fig. 5g). These results show that Arp2/3-dependent actin nucleation around the nucleus is required to pass through small constrictions only in lamin A/C-expressing cells and they suggest that its function might be to facilitate nuclear deformation through transient rupture or disassembly of the lamin A/C network.

The requirement for a branched actin network (nucleated by Arp2/3 rather than formins) might be linked to the capacity of such networks to exert strong pressure via an increase in the density of branches when growing under a load or in confinement^39^,^40^,^41^,^42^,^43^. Building on this concept, we propose that the accumulation of actin around the nucleus could be simply induced by the high load produced by the stiff nuclear lamin A/C mesh when it is confined within the constriction. Consistent with this proposal, we found that actin filament accumulation around the passing nuclei was reduced in cells depleted for lamin A/C (Fig. 5h,i). This suggests that the presence of the nuclear lamina not only makes Arp2/3 and perinuclear actin essential for nuclear passage, but is also responsible for triggering the formation of the perinuclear actin network on nuclear passage through a constriction.

### CiAN does not depend on the LINC or on the nucleus

**Figure 6:**
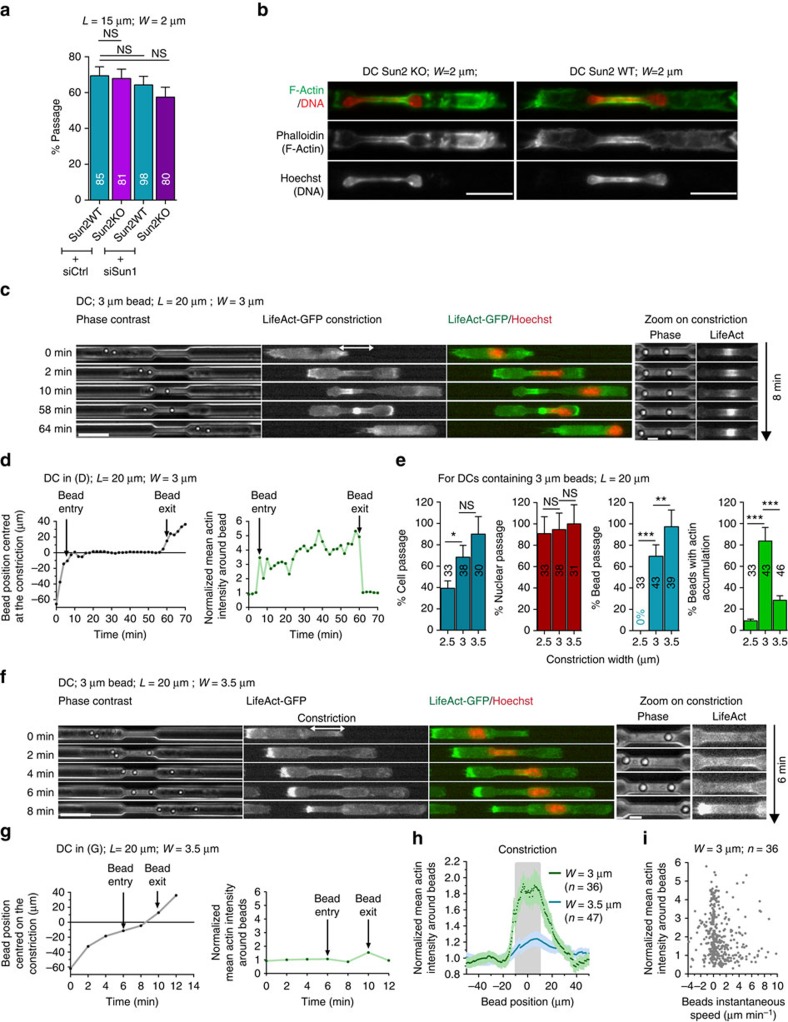
Actin assembly inside constrictions is not specific to the nucleus and can be induced by confinement of rigid particles. (**a**) Percentage of passage in 2-μm-wide constrictions of DCs treated as indicated. Error bars are s.e.m. (**b**) Representative images of Sun2 KO (left) and Sun 2 WT (right) immunostained DCs in 2-μm-wide and 15-μm-long constrictions. *N*=2; scale bar, 15 μm. Sequential images of representative LifeAct-GFP (green)-expressing DCs with a 3 μm internalized bead stained with Hoechst (red) passing 3 μm (**c**) and 3.5 μm (**f**) wide constrictions. Scale bar 20 μm (**c**,**f** (left)); 5 μm (zoom). (**d**) Left: bead position centred at the constriction; (**d**) right: normalized mean actin intensity around the first bead of the cell shown in **c** over time. (**e**) For DCs with internalized beads, percentage of cells with F-actin accumulation around beads (‘Peribead'), percentage of cell, bead and nuclear passage as function of the constriction width (2.5, 3 and 3.5 μm). Numbers represent the number of cells observed for *N*=3. Error bars are s.e.m. (**g**) Left: bead position centred at the constriction; (**g**) right: normalized mean actin intensity around the third bead of the cell shown in **f** over time. (**h**) Normalized mean actin intensity around beads in 3- (green) and 3.5-μm (blue)- wide constrictions as a function of the bead centre of mass position relative to the centre of the constriction. Error bars are s.e.m. (**i**) Normalized mean actin intensity around beads as function of beads velocity in 3-μm-wide constrictions. *L*, constriction length; *W*, constriction width.. ***P-value<0.0001, **P-value<0.001 and *P-value<0.01. NS, nonsignificant. Statistical test: Fisher's test.

To test whether actin accumulation was due to a specific pathway associated with the presence of the nucleus in the constriction, we performed two sets of experiments. First, we inhibited the LINC complex, which is known to physically link the nuclear periphery to the actin cytoskeleton (for a recent review, see ref. 44). We chose to deplete the SUN proteins^45^,^46^. We used cells from a SUN2KO mice^47^ and RNA interference depletion of Sun1 (Supplementary Fig. 13A,B). As expected, we observed that LINC-depleted cells were less persistent and, consistently, have a lower non-passage time (Supplementary Fig. 13C–E). Nevertheless, we did not observe any effect on the percentage of cell passage through 2-μm-wide constrictions (Fig. 6a and Supplementary Fig. 13F) and actin accumulation was still observed around the passing nuclei (Fig. 6b representative of 16 over 20 cells, *N*=2). This suggests that the LINC complex is necessary neither for nuclear passage nor for the induction of actin polymerization on nuclear entry in the constriction.

Second, we reasoned that if actin nucleation was simply induced by confinement of the Arp2/3-nucleated network by the stiff nucleus, any stiff object inside the cell should have the same effect. We took advantage of the capacity of DCs to internalize large particles^24^ and incubated cells with polystyrene beads of 3 μm of diameter. The migration of LifeAct-GFP-expressing DCs loaded with such beads through 3.5-, 3- and 2.5-μm-wide constrictions was then recorded (Supplementary Movie 12). In more than 80% of cases, actin accumulated around 3 μm beads when they passed through 3 μm constrictions (Fig. 6c–e and Supplementary Movie 12). In 2.5 μm constriction, no beads could pass (beads remained stuck at the constriction entry) and no actin was observed around these beads, showing that entry inside the constriction was required to trigger actin accumulation (Fig. 6e). Although in this range of sizes (2.5–3.5 μm) the rate of nuclear passage did not change, the rate of cell passage was correlated to the rate of bead passage through the constrictions (Fig. 6e), showing that beads were then the limiting factor for cell passage. In 3.5 μm constrictions, no actin accumulation was observed around the beads (Fig. 6e–h). No correlation was observed between bead velocity and the measured actin density around beads, ruling out a requirement for shear stress on actin accumulation in constrictions (Fig. 6i). Moreover, when cells were loaded with both 2 μm and 3 μm beads, actin accumulation was observed only around 3 μm beads that entered the 3 μm wide constrictions (Supplementary Movie 13). These experiments show that stiff objects that are confined in constrictions, that is, beads that match the constriction size, can induce the accumulation of actin to the same extent as the nucleus. Together, these data strongly suggest that actin accumulation around the nucleus in constrictions is caused by confinement due to nuclear stiffness and rule out the requirement for specific nucleus-associated components.

## Discussion

Our results identify a new mechanism required for the migration of fast-moving lamin A/C-expressing cells through small constrictions. This mechanism involves the nucleation of an Arp2/3-dependent actin network around the nucleus, which would transiently weaken the lamin A/C nuclear shell, making the nucleus more deformable. A transient rupture of the lamin A/C intranuclear shell, followed by re-assembly, has already been documented in the case of an imposed nuclear deformation^37^, suggesting that strong pushing forces could rupture the nuclear lamina without killing the cell. Arp2/3 actin nucleation was also found to facilitate the nuclear envelope rupture at mitotic entry in starfish oocytes^48^, a phenomenon also associated with nuclear lamina disassembly. The precise mechanism by which actin assembly is triggered and the nuclear lamina shell deformed will require further study (see the Supplementary Discussion section on other actin structures recently reported to be associated to the cell nucleus^49^,^50^,^51^,^52^,^53^,^54^,^55^).

The LINC complex, which binds both the nuclear lamina and the cytoskeleton, is not required for nuclear passage through micrometric pores (see also the Supplementary Discussion section). As shown for other processes such as nuclear envelope breakdown^56^ and the adaptation of nuclear stiffness to tissue mechanical properties^38^, lamin A/C assembly might be regulated by phosphorylation on nuclear deformation. A recent article indeed suggested that ATR, a kinase normally involved in DNA repair, was activated and localized to the nuclear periphery on mechanical perturbation of the nucleus^57^. Nevertheless, our observation of very local ruptures of the lamin A/C shell rather suggests a mechanical rupture than a signalling event.

According to our results, other cell types that do not express lamin A/C and thus have a softer nucleus should not show perinuclear actin accumulation. To test this idea, we performed a similar set of experiments on HL60-derived neutrophils, which have a low lamin A/C expression level^20^ (Supplementary Fig. 1A,B). These cells could pass through 1 μm constrictions (Supplementary Fig. 14A–C), which WT DCs cannot pass, consistent with a softer nucleus^20^. Similar to DCs depleted in lamin A/C, they did not need Arp2/3 to pass small constrictions (Supplementary Fig. 14D,E). No actin accumulation was observed around the nucleus of HL60-derived neutrophils even in the smallest constrictions (Supplementary Fig. 14A,F), with actin being rather enriched at the cell back (Supplementary Fig. 14A). Overall, these results confirmed that Arp2/3-dependent actin nucleation around the nucleus is required only for the deformation of nuclei, which possess a lamin A/C network. They also confirm that accumulation of perinuclear actin filaments depends on the presence of a lamin A/C intranuclear shell.

A surprising result from our study is that myosin II, while increasing the speed of cells through constrictions, was not required for the passage itself. This was also the case for cells after lamin A/C knockdown (Fig. 5f) and for neutrophils when passing similar constriction sizes (Supplementary Fig. 14G,H). Nevertheless, myosin II activity was required for neutrophils to pass through the smallest constrictions (1 μm), DCs not being able to pass such constriction (Supplementary Fig. 14D,E,G,H). In this case, in the absence of a nuclear lamina shell the resisting element might be the chromatin itself, which is known to have a high viscosity^58^. myosin II contractility at the cell rear could then become necessary, to produce enough pressure to allow a flow of this viscous material through such a narrow pore. The mechanism we describe here allows fast migration through small constriction, by combining myosin II-dependant rear contraction and Arp2/3-dependant lateral compression of the nucleus (see the Supplementary Discussion section on the difference between mesenchymal and amoeboid cells).

Altogether, our results are in qualitative agreement with the following model (Supplementary Fig. 15): when cells face small constrictions, forces generated by the migration apparatus are enough to push the nucleus against the constriction, leading to the insertion of a part of the nucleus, whose size depends on the constriction width, the nuclear stiffness and the pushing force. The part of the nucleus inserted in the constrictions triggers the accumulation of Arp2/3-nucleated actin, due to confinement of the network^43^,^59^. This actin network produces a lateral compression force, which further deforms the part of the nucleus inserted in the constriction, allowing further entry. When nuclear surface deformation reaches a certain threshold, the nuclear lamina locally ruptures, allowing full passage of the nucleus, at a rate set by the pushing force, the nuclear viscosity and the constriction cross-section area. In-depth physical modelling and quantitative biophysical experiments will be necessary to further test this model.

The fact that CK666 treatment reduces the percentage of cells that pass through the constrictions (Fig. 2a) but not the passage time (Fig. 2b) might seem surprising. Nevertheless, in the frame of our working model, this is perfectly consistent. The lateral compression by the Arp2/3-nucleated actin does not produce any forward pushing force; it is fighting against the energy barrier set by the stiffness of the elastic nuclear lamina. Thus, this contribution of Arp2/3-nucleated actin sets the level of deformation that can be reached. On the contrary, the cortex tension imposed by myosin II motors sets the difference of pressure between the front and the back of the nucleus when it is obstructing the constriction. This difference of pressure sets the time it will take, for a given viscosity, to pass through a constriction of a given size. In conclusion, myosin II motor activity is related to the viscous part of the nuclear resistance and this sets the passage time for a given channel size, whereas Arp2/3-nucleated actin is related to the elastic part of the nuclear resistance and thus sets the fraction of passing cells for a given amount of deformation of the nuclear surface required for passage. Importantly, such reasoning also suggests that depending on the visco-elastic properties of their nucleus and their migration speed, various types of cells might fall either in a range of parameters in which the elastic properties of their nucleus dominates, making Arp2/3-nucleated actin essential, or in a range of parameters in which the nucleus is rather viscous, in which case Arp2/3 will not be required and myosin II activity will set the passage time. As most visco-elastic materials are viscous at long time scales, a prediction of that model is that slow migrating cells, just similar to cells that do not express lamin A/C, will not require Arp2/3 for nuclear passage.

In conclusion, we show that DCs possess a specific mechanism, based on Wave2/Arp2/3 actin nucleation around the nucleus that enables them to deform their nucleus despite the presence of a stiff lamin A/C intranuclear shell. We propose that this mechanism helps them combine a high migration speed and a high level of deformability, with long-term survival. These characteristics are required for DCs to achieve their functions from peripheral tissue sampling to antigen presentation in lymph nodes. We anticipate that this mechanism might be used by other long-lasting leukocytes, and that it could facilitate the migration and survival of fast amoeboid metastatic cells through dense matrices and tissues.

## Methods

### Mice

Myosin IIA knockout mice were generated by crossing MyoIIAflox/flox mice^10^ with CD11c-Cre mice^60^ and with Gt(ROSA)26Sor–flox-stop-flox–YFP mice (Rosa-YFP)^61^, to obtain MyoIIAflox/flox-CD11c-Cre+-Rosa-YFP mice that were used as bone marrow donors. Littermates or age-matched MyoIIAwt/wt-CD11c-Cre+–Rosa-YFP mice were used as control bone marrow donor mice. The breeder mice were previously backcrossed to C57BL/6 for at least seven generations.

I-Aβ^b^-GFP and Myosin IIA-GFP knock-in mice were previously described^36^,^62^.

Lifeact-GFP mice were a kind gift from Michael Sixt lab (IST, Austria) and were generated as described^34^.

Details of the generation of the Hem1 knockout mice are described in ref. 33. Briefly, exons 4 and 5 were targeted and flanked with loxP sites and deletion of Hem1 was induced by crossing the mouse with a mouse expessing Cre recombinase under control of the keratin 14 promoter. Cells from Hem1KO mice (Stradal Lab) were kindly provided by Michael Sixt.

mDia1 knockout mice were obtained from Arthur Alberts lab (Van Andel Institute, USA) and were generated as described in ref. 63.

Sun 2 knockout mice previously described in ref. 47 were obtained from Megan King's lab (Yale School of Medicine, USA).

C57BL/6 mice, described in ref. 25, were used for immunofluorescence (in Figs 4c and 5a,b, and Supplementary Figs 3A,5D,6A and 11A). Each result presented here was obtained from two to four independent experiments (two to four different mice). The experiments were performed on 6-week-old male or female mice. For animal care, we strictly followed the European and French National Regulation for the Protection of Vertebrate Animals used for Experimental and other Scientific Purposes (Directive 2010/63; French Decree 2013-118). The present experiments, which used mouse strains displaying non-harmful phenotypes, did not require a project authorization and benefited from guidance of the Animal Welfare Body, Research Centre, Institut Curie.

### Cells

Immature mouse bone-marrow-derived DCs were cultured 10–12 days in DC medium (IMDM, FCS (10%), Glutamine (20 mM), pen–strep (100 U ml^−1^) and 2-ME (50 μM)) supplemented with granulocyte–macrophage colony stimulating factor (50 ng ml^−1^)-containing supernatant obtained from transfected J558 cells, as previously described^25^.

To generate neutrophils, HL60 cells were maintained in culture and differentiated with 1.3% of DMSO as previously described in ref. 64. Lyn-emerald/Utrophin-mCherry HL60 was a kind gift from Dyche Mullins lab (UCSF, USA) and actin-GFP HL60 was kindly provided by Guillaume Charras (UCL, UK).

### Antibodies and reagents

For imaging the nucleus, cells were incubated with 200 ng ml^−1^ of Hoechst 33342 (Life Technologies) or 34580 (Invitrogen) for 30 min at 37 °C and 5% CO_2_.

The following antibodies were used for immunoblotting: LmnA/C (H110, Santa Cruz), anti-ArpC4 and anti-sun2 (Abcam), and anti-actin (Millipore). Antibodies were used at 1:1,000 dilution.

For immunofluorescence, we used Alexa Fluor 594-coupled phalloidin (Invitrogen; 1:400), anti-LmnAC (N18, Santa Cruz (1:50) and clone 4C11 from Sigma Aldrich (1:2,000)), anti-Lamin B1 (ab16048, Abcam (1:500)) and anti-Arp3 (Abcam; 1:200); DAPI (4,6-diamidino-2-phenylindole) for DNA staining; and secondary antibodies anti-mouse-Alexa488 and anti-Goat-Alexa488 from Jackson ImmunoResearch Laboratories were used at 1:400. Slides were mounted with custom-made Moviol.

For lymphatic vessels visualization, anti-LYVE-1 (R&D System, 1/1,000) was used.

For drug treatment CK666, latrunculin A and blebbistatin were obtained from Tocris Bioscience, Y27632 from Calbiochem, nocodazole and DMSO from Sigma-Aldrich. For silencing, Smart pool ON-TARGETplus mouse LMNA siRNA, Arpc4 siRNA and Sun1 siRNA were purchased from Thermo Fisher Scientific. Micro-channels were coated with fibronectin (Sigma) or custom-made pLL(20)-g[3.5]-PEG(2)-Rhodamin.

### siRNA silencing

DCs were transfected with SMARTpool: ON-TARGETplus siRNAs specific for Arpc4, LMNA or Sun1 (Thermo scientific) using the Amaxa Mouse Dendritic Cell Nucleofector Kit (Lonza). Briefly, 3 × 10^6^ DCs collected at day 7 of differentiation were transfected in 100 μl of Amaxa solution containing 1 μM of the desired siRNA. Transfected cells were cultured during 48–72 h in DC medium and then used to perform the desired experiment.

We noted that systematically, and for unknown reasons, control siRNA-electroporated DCs migrate faster than non-electroporated ones. Thus, they have a higher rate of passage through constrictions and a lower passage time (Supplementary Fig. 2 compared with Supplementary Fig. 12). For this reason, values should be compared with their internal control (either non-electroporated or electroporated).

### Beads internalization

DCs were concentrated at 2 × 10^7^ cells per ml and incubated with 3 μm polystyrene beads (17145-5, amino beads, Polysciences) at 2:1 beads per cells ratio for 1 h at 37 °C/5% CO_2_. Cells were resuspended in 1 ml of PBS (Gibco), which is then deposited on 3 ml of FCS before being centrifuged at 900 r.p.m. for 5 min, allowing the removal of floating non-internalized beads.

### Channels preparation and cell loading

Microchannels were prepared as previously described^25^,^28^. Briefly, PDMS (GE Silicones) was used to prepare 7 × 5 μm microchannels with constrictions from a custom-made mould. The PDMS chamber and a 35-mm glass-bottom dish (FD35-100, WPI) were plasma activated before being bound to each other. The binding was left to strengthen in a 65 °C oven for 1 h. The so-obtained microchannels were plasma cleaned then incubated with 50 μg ml^−1^ of fibronectin or 100 μg ml^−1^ of pLL-PEG-Rhodamin at RT for 1 h then washed with PBS before being incubated with the adequate medium (containing drugs if necessary) for at least 1 h at 37 °C and 5% CO_2_ before cell loading.

### Drugs treatment

CK666 was used at 50 μM, latrunculin A at 50, 100 and 250 nM, blebbistatin at 50 μM, Y27632 at 30 μM for DCs and 10 μM for HL60-derived neutrophils, nocodazole at 10 μM and DMSO was used as control for all drugs, except Y27632, which was dissolved in water. When drugs were used in microchannel experiments, the chamber was incubated with drug containing medium for at least 1 h before cell loading. Cells were resuspended in drug containing medium before being deposited in the chamber entry pores.

### Immunoblotting

DCs were lysed on ice for 45–60 min in a buffer containing either 50 mM Tris-HCl, 150 mM NaCl, 1% NP-40, 1% sodium deoxycholate and 0.1% SDS (Supplementary Fig. 1A,B) or 100 mM Tris, 150 mM NaCl, 0.5% NP-40 (Supplementary Figs 5,12 and 13) or 1:100 of protease inhibitor cocktail (Roche) and 1:100 of phosphatase inhibitor cocktail (Sigma). Protein concentration was quantified using the DC Protein Assay (Biorad). Cell lysate was suspended in 2 × loading buffer containing 4% SDS. Thirty micrograms of soluble extracts were loaded onto a 4–20%/4–12% TGX gradient gel (BioRad) and transferred onto a Trans-Blot Turbo PVDF membrane (BioRad). The membrane was blocked using in 1 × Tris-Buffered saline solution containing 0.1% of Tween-20 and 5% of Milk, incubated with the appropriate antibodies and revealed with SuperSignal West Dura substrate (Thermo Scientific).

### Quantitative PCR

Cells were cultured as described above. RNA extractions were performed using NucleoSpin RNA (Macherey-Nagel), according to manufacturer's protocol. Complementary DNA were obtained with SuperScriptVILO cDNA synthesis kit (Life Technologies), according to manufacturer's protocol, starting from 1 μg of RNA. Quantitative PCR were done with the Lightcycler 480 (Roche) using Taqman Gene expression assay (Applied Biosystem) with the following primers: Mm00659179_m1 (best coverage primers for mouse Sun1) and Mm99999915_g1 (gapdh, as a control).

### Video microscopy and quantification of cell passage through constrictions

Migrating cells were imaged for 15–20 h on an epifluorescence microscope (Nikon TiE) equipped with a cooled CCD (charge-coupled device) camera (HQ2, Photometrics), with a × 10, 0.3 or 0.5 numerical aperture (NA) objective. We recorded an image every 2 min. When blebbistatin was not used, nuclear position was determined by recording the Hoechst signal with the same temporal resolution. We focused on the passage of the first constriction encountered by the cell. Characteristics of cell passage through constrictions were determined using a custom-made semi-automatized ImageJ macro combined with a Matlab script. The percentage of passage was calculated as the ratio between the number of cells that passed a constriction and the number of cells that encountered a constriction. The cell (or nucleus) passage time represents the time between the cell (nucleus) entering the constriction and the cell (nucleus) back exiting the constriction. The cell non-passage time corresponds to the time spent by the cell in the constriction before changing direction and moving backward.

### Cell tracking

LifeAct and Hoechst signals were used to generate the mask of the cell and the nucleus, respectively. A custom-made ImageJ macro combined with a Matlab script was used to analyse cell and nuclear trajectories. Briefly, positions of the cell and nuclear centre of mass and edges (front and back) were detected from the masks at each time point. The change of speed between the channel and the constriction was determined by subtracting, at each time point, the mean cell velocity before the nucleus touched the constriction to the instantaneous speed in the constriction. The normalized velocity variation was then calculated as the ratio between the velocity variation and the mean velocity before the constriction, for each cell.

### Quantification of actin enrichment around the nucleus and beads

Hoechst stained DCs from LifeAct-GFP mice or actin-GFP overexpressing HL60-derived neutrophils were loaded in microchannels and imaged using an epifluorescence microscope (Nikon TiE) equipped with a cooled CCD camera (HQ2, Photometrics) and a × 20 dry objective with a high NA (Nikon × 20 0.75 NA DIC or phase contrast). The LifeAct and Hoechst signals were used to generate the mask of the cell and the nucleus, respectively. A custom-made ImageJ macro combined to a Matlab (version R2011a) script was used to analyse the actin dynamics during cell passage through constrictions. The mean actin in constrictions was defined as the ratio between the total LifeAct signal and the cell area in the constriction. This value divided by the mean LifeAct intensity in the cell gave the normalized mean actin intensity inside constrictions. To obtain the normalized mean actin intensity around the nucleus, the mean LifeAct intensity in the mask of the cell cortex around the nucleus was determined and normalized by the mean LifeAct intensity in the whole cell. The mask of the cell cortex was obtained by subtracting a six-times-eroded from a one-time-dilated mask of the cell.

The same method was used to quantify actin dynamics around internalized beads. Beads masks were obtained by hand drawing of beads contours.

### Immunofluorescence inside microchannels

To perform immunostaining on cells migrating inside microchannels, the PDMS chamber was reversibly bound to the glass coverslip by plasma activating only the glass coverslip. DCs were allowed to migrate in microchannels for 16 h. Cells were then fixed with 4% paraformaldehyde for 30 min at room temperature. After three washes with PBS, the PDMS was carefully removed to insure cell reachability during immunostaining. Cells were then permeabilized with PBS containing 0.5% Triton for 5 min and rinsed with PBS–5%BSA for 1 h at room temperature. Cells were incubated with primary, then secondary antibodies diluted in PBS–1%BSA. Phalloidin and DAPI staining was performed during the secondary antibody staining. Coverslips were washed with PBS and mounted in custom-made mounting media (Moviol). Image acquisition was performed on an inverted epifluorescence microscope (Nikon TiE) equipped with a cooled CCD camera (HQ2, Photometrics) and a × 100, 1.4 NA oil-immersion objective.

### Actin visualization inside collagen gels

Collagen gels were prepared at 3.0 mg ml^−1^ of Rat-tail collagen (10 mg ml^−1^, Ibidi). DCs were embedded in polymerizing collagen at a concentration of 2.10^6^ ml^−1^ and then placed on glass-bottom dishes. After 20 min of collagen gelation at 37 °C, medium was added to equilibrate the gel and cells were fixed with 4% paraformaldehyde for 40 min before being stained with phalloidin.

### Actin visualization in ear explants

Ears from C57BL/6 mice were excised and a pair of forceps was used to create a hole.The ventral and dorsal sides of the explant were separated by peeling. The ventral sheet was kept and immunostained with anti-LYVE-1 (R&D Systems) primary antibody, to mark the lymphatic vessels. After washing with media, a secondary antibody against rat (Jackson Immunoresearch, 712-166-150) was used. The ear sheet was then spread flat in a six-well plate and a PDMS block with a central hole of diameter 8 mm was placed on top of each explant. Two hundred thousand LifeAct-GFP-expressing DCs labelled with Hoechst were added in 100 μl of culture medium inside the hole. After 1 h of incubation, the ear sheet was washed with culture medium and then placed with the face on which cells were incubating against the bottom glass slide in a FluoroDish. A block of PDMS was added on top to prevent the explant from moving during imaging. Imaging was performed on an inverted confocal microscope, at 37 °C and with 5% CO_2_.

### Actin visualization in medaka fish

Imaging was performed on TG(FmpoP::EB3-EGFP/FmpoP::RFP-LifeAct)^65^ transiently expressing the nuclear H2B-CFP marker at 9 days post fertilization, wounded and mounted as previously reported^66^. Time-lapse fluorescence images were acquired with an UltraView VoX spinning disk confocal unit (Perkin Elmer) equipped with an inverted Nikon Eclipse Ti microscope, coupled to a C9100-50 emCCD camera (Hamamatsu) and a Yokogawa CSU-X1 scanning head, and driven by Volocity software (Improvision, Perkin-Elmer). Cyan fluorescent protein/green fluorescent protein (GFP)/red fluorescent protein (RFP) imaging was performed using 405, 488 and 568 nm laser lines with a specific multiband-pass emission filter. Image sequences were acquired at 15–18 s time resolution using, an NA 1.3/ × 40 oil-immersion objective lens and 1 μm step size. Multi-channel images were acquired before switching *Z*-planes to improve the accuracy of co-localization between channels.

### Statistics

GraphPad prism software was used to produce all the graphs. We used the Mann–Whitney test and the Fisher's test, to determine the significance between two distributions of respectively random and Boolean variables. Mann–Whitney tests were performed using GraphPad prism and Fischer's tests were performed using using R (version 3.0.2).

### Code availability

Codes used to analyse cell passage through constrictions and actin dynamics during cell migration are accessible on request.

## Additional Information

**How to cite this article:** Thiam, H. R. *et al.* Perinuclear Arp2/3-driven actin polymerization enables nuclear deformation to facilitate cell migration through complex environments. *Nat. Commun.* 7:10997 doi: 10.1038/ncomms10997 (2016).

## Supplementary Material

Supplementary Figures, Supplementary Figures 1-15, Supplementary Tables 1-2, Supplementary Discussion and Supplementary References

Supplementary Movie 1 Phase contrast time-lapse imaging of DCs migrating through 20 μm long constrictions of various cross-sectional areas (23 μm^2^ = 5 μm x 4.6 μm; 4.5 μm^2^ = 1.5 μm x 3 μm; 2.2 μm^2^ = 1 μm x 2.2 μm). Hoechst staining (green) of DNA allowed nuclear localization. Time in hour: minutes. Each movie is followed by a zoom on a single cell.

Supplementary Movie 2 Low resolution (20X binning 2) time-lapse imaging of LifeAct-GFP expressing DCs migrating on a 2D glass coverslip. Cells were treated with DMSO or 50, 100, 250 nM of Latrunculin A. Hoechst staining (red) of DNA allowed nuclear localization. Time in hour: minutes.

Supplementary Movie 3 High resolution (60X, binning 2), time-lapse imaging of a LifeAct-GFP (green) expressing DC migrating through a 15 μm long, 7 μm^2^ (2 μm x 3.5 μm) constrictions. Hoechst staining (red) of DNA allowed nuclear localization. Time in hour: minutes.

Supplementary Movie 4 Low resolution (20X binning 1), time-lapse imaging of LifeAct-GFP (green) expressing DCs migrating through 20 μm long constrictions of various cross-sectional areas (23 μm^2^ = 5 μm x 4.8 μm; 12 μm^2^ = 3 μm x 4 μm. 4.5 μm^2^ = 1.5 μm x 3 μm; 2.2 μm^2^ = 1 μm x 2.2 μm). Hoechst staining (red) of DNA allowed nuclear localization. Time in hour: minutes. Each movie is followed by a zoom on a single cell.

Supplementary Movie 5 Time-lapse imaging of LifeAct-GFP (green) expressing dendritic cell migrating through 15 μm long, 7 μm^2^ (2 μm x 3.5 μm) constrictions. Hoechst staining (red) of DNA allowed nuclear localization. Channels are coated with PLL-PEG to prevent the formation of adhesion complexes. Time in hour: minutes. Movies of single channels are concatenated for better visibility.

Supplementary Movie 6 Time-lape imaging of LifeAct-GFP (green) expressing DCs migrating in a mouse ear explant. Hoechst staining (red) of DNA allowed nuclear localization. Lymph vessels were stained with a rat-anti-Lyve-1 antibody (blue). Time in minutes: seconds. The movie is followed by a zoom on a single cell then by a rotated view of this cell.

Supplementary Movie 7 Time-lapse imaging of RFP-LifeAct (actin, green) and H2B-CFP (chromatin, red) expressing myeloid cells migrating in mekada fish after wounding of the tail. The movie shows two representative cells. Time in hour: minutes: seconds. White arrows indicate the presence of an actin accumulation.

Supplementary Movie 8 Time-lapse imaging of an invariant chain knock-out dendritic cell migrating through a 15 μm long, 7 μm^2^ (2 μm x 3.5 μm) constriction. Hoechst staining (red) of DNA allowed nuclear localization. Reflection interference contrast microscopy was used to visualize cell-substrate contact areas. Time in hour: minutes.

Supplementary Movie 9 Low resolution (20X binning 2) time-lapse imaging of LifeAct-GFP expressing DCs migrating through 15 μm long, 7 μm^2^ (2 μm x 3.5 μm) constrictions. Cells were treated with DMSO or 50nM of Latrunculin A. Hoechst staining (red) of DNA allowed nuclear localization. Time in hour: minutes.

Supplementary Movie 10 Time-lapse imaging of LifeAct-GFP (green) expressing DCs migrating through 15 μm long, 7 μm^2^ (2 μm x 3.5 μm) constrictions. Cells were treated with DMSO or with 50 μM CK666. Hoechst staining (red) of DNA allowed nuclear localization. Time in hour: minutes. The first movie is followed by a zoom on a single passing cell. The second movie is followed by a zoom on a single non-passing cell, then by a zoom on a passing cell.

Supplementary Movie 11 Myosin IIA-GFP (green) expressing DCs migrating through 15 μm long, 7 μm^2^ (2 μm x 3.5 μm) constrictions. Hoechst staining (red) of DNA allowed nuclear localization. Time in hour: minutes. The movie is followed by a zoom on a single cell.

Supplementary Movie 12 Low resolution (20X binning 1), time-lapse imaging of LifeAct-GFP (green) expressing DCs migrating through 20 μm long constrictions of various cross-sectional areas (10 μm^2^ = 2.5 μm x 4 μm; 12.6 μm^2^ = 3 μm x 4.2 μm; 14.7 μm^2^ = 3.5 μm x 4.2 μm). Bright spots in phase contrast represent the internalized beads. Hoechst staining (red) of DNA allowed nuclear localization. Time in hour: minutes.

Supplementary Movie 13 Low resolution (20X binning 2), time-lapse imaging of LifeAct-GFP (green) expressing DCs migrating through 3 μm wide and 20 μm long constrictions. DCs have internalized beads of 3 μm and 2 μm of diameter. Bright spots in phase contrast represent the internalized 3 μm beads while 2 μm beads are in cyan. Hoechst staining (red) of DNA allowed nuclear localization. Time in hour: minutes. White arrows indicate the position of 2 μm beads. Time in hour: minutes.
