# Supplementary material for: Perinuclear Arp2/3-driven actin polymerization enables nuclear deformation to facilitate cell migration through complex environments
Source: Nat Commun. 2016 Mar 15;7:10997. doi: 10.1038/ncomms10997 (PMC4796365; doi:10.1038/ncomms10997)
Supplement: Supplementary Figures, — Supplementary Figures 1-15, Supplementary Tables 1-2, Supplementary Discussion and Supplementary References [file ncomms10997-s1.pdf]

Supplementary Figure 1

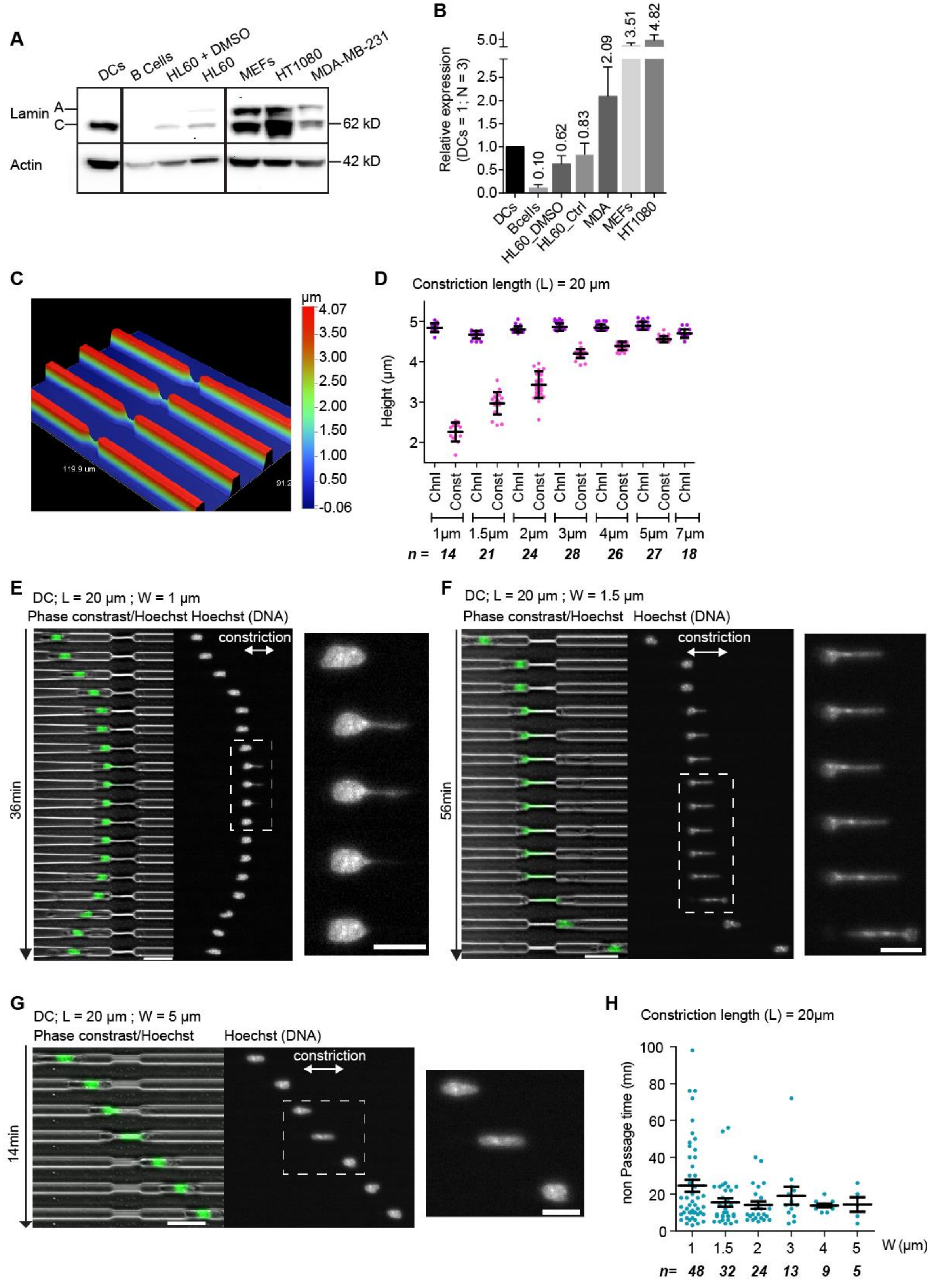

Supplementary Figure 1: Characterization of constriction sizes and details of DCs migration through 20 µm long constrictions. (**A**) Western blot showing lamin A/C expression level in various representative cell types for a qualitative comparison of typical cell types used in 3D cell migration studies and characterized for their lamin A/C expression, with mice bone-marrow derived DCs, which have not been characterized before for their level of lamin A/C expression; G-actin is shown as loading control. (**B**) Quantification from 3 independent western blots of the relative lamin A/C expression in cells in (A). Bars represent the mean; error bars the standard error on the mean. (**C**) Image from an optical profilometer of a region of a master mold containing channels with constrictions. (**D**) Measure of channels and constrictions height by confocal microscopy. Lines show means; bars show standard deviations. Chnl = channels, Const = constriction. The width of the constrictions is shown on the X axis. (**E**) Sequential images of a DC stained with Hoechst (DNA, green) attempting to migrate through a 1 µm wide constriction. (**F**) and (**G**) Sequential images of DCs migrating through respectively 1.5 µm and 5 µm wide constrictions. (**E**), (**F**) and (**G**) Right side: magnified images to visualize nuclear deformation. Scale bar, 30 µm for phase contrast and DNA overlays, 15 µm for zoom of the DNA in constrictions. (**H**) Quantification of the time spent by non-passing cells in constrictions as a function of the constriction cross section area. The mean nonpassing time (> 14 minutes) is much higher than zero, meaning that cells which did not pass constrictions did not turn back because of a lack of persistence. (**D**) and (**H**) n represents respectively the number of channels and cells. W: constriction width.L: constriction length. Error bars in (**H**) represent standard deviation.

Supplementary Figure 2

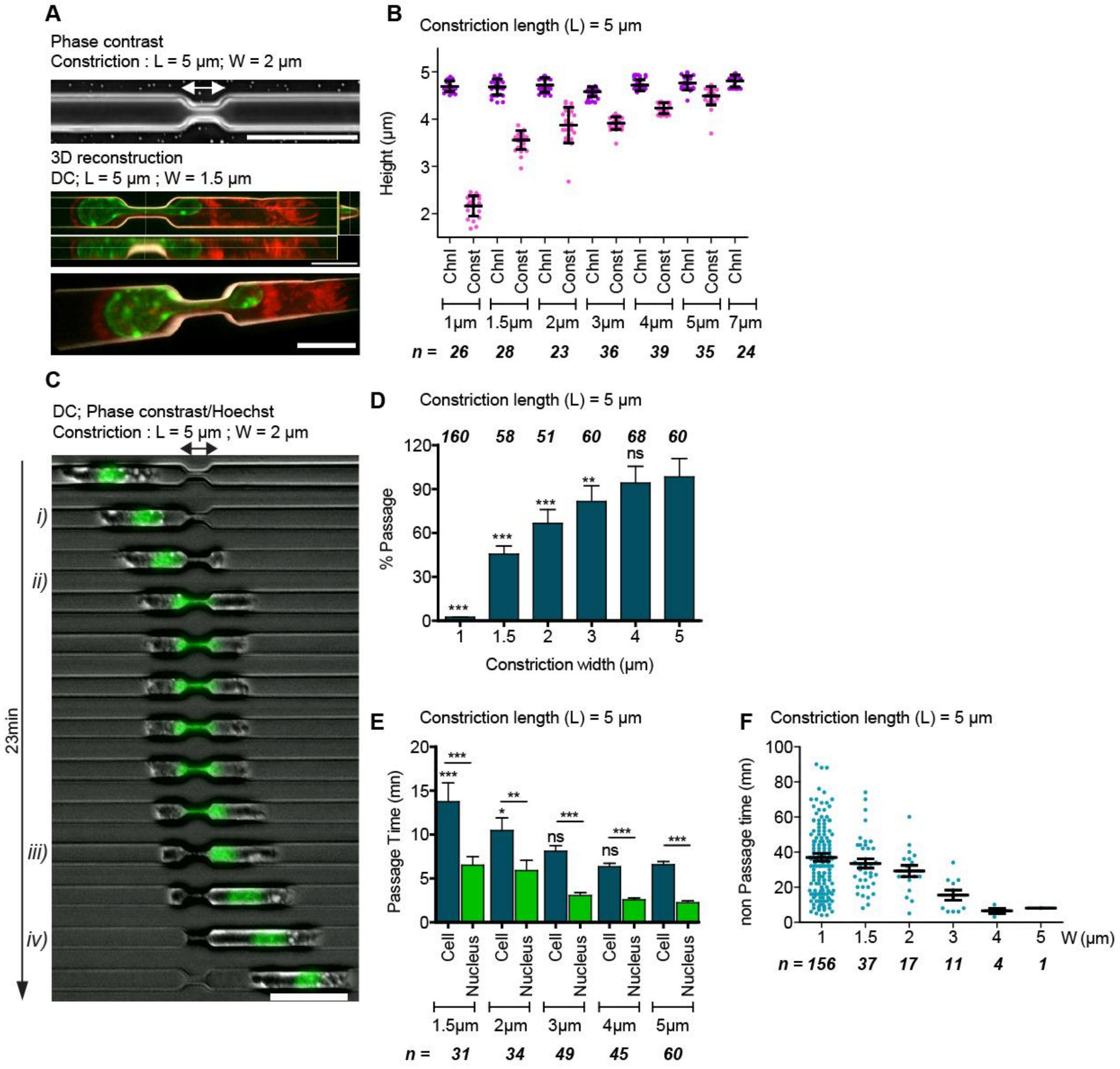

Supplementary Figure 2: The physical limit imposed by the nucleus is independent of the constriction length: characterization of the 5 µm long constrictions. (**A**) Top: phase contrast image of a channel with a 5 µm long and 2 µm wide constriction. Middle and bottom: 3D reconstruction from a 3D stack. Iaβ-GFP (red) DC in a 1.5 µm wide constriction coated with pLL-PEG (gray scale). The DNA in green is stained with Hoechst. Middle: Different views of the 3D reconstruction. Right: view of the cross section. Bottom: Perspective view of the 3D reconstruction with a 45° angle.

Scale bar top: 30 µm, middle and bottom: 10 µm. (**B**) Measure of channels and constrictions height by confocal microscopy. Lines for mean; bars for standard deviation. Chnl for channels, Const for constriction. The width of the constrictions is shown on the X axis. n represents the number of channels (**C**) Sequential images of an DC crossing a 2 µm wide constriction. i), ii), iii), iv) respectively indicate cell entry, nuclear entry, nuclear exit, cell exit. Scale bar 30 µm. (**D**) Percentage of passage as function of the constriction cross section area. Numbers above bars represent the number of cells scored. Time in minutes: seconds. (**E**) Cell (blue) and nuclear (green) passage time. Unless when indicated by a spanner, statistical test was against the value for 5 µm wide constrictions. P-value: ***, ** and * indicate < 0.0001, < 0.001, and < 0.01. ns = non-significant. Statistical test: Fisher test for (**D**), Mann-Whitney test for (**E**). (**F**) Cell non passage time which measures the time spent in the constriction by non-passing cells. (**E**), (**F**): n represents the number of cells scored. W: constriction width. Error bars in (**D**) and (**E**) represent standard deviation. Error bars in (**F**) represent standard deviation

Supplementary Figure 3

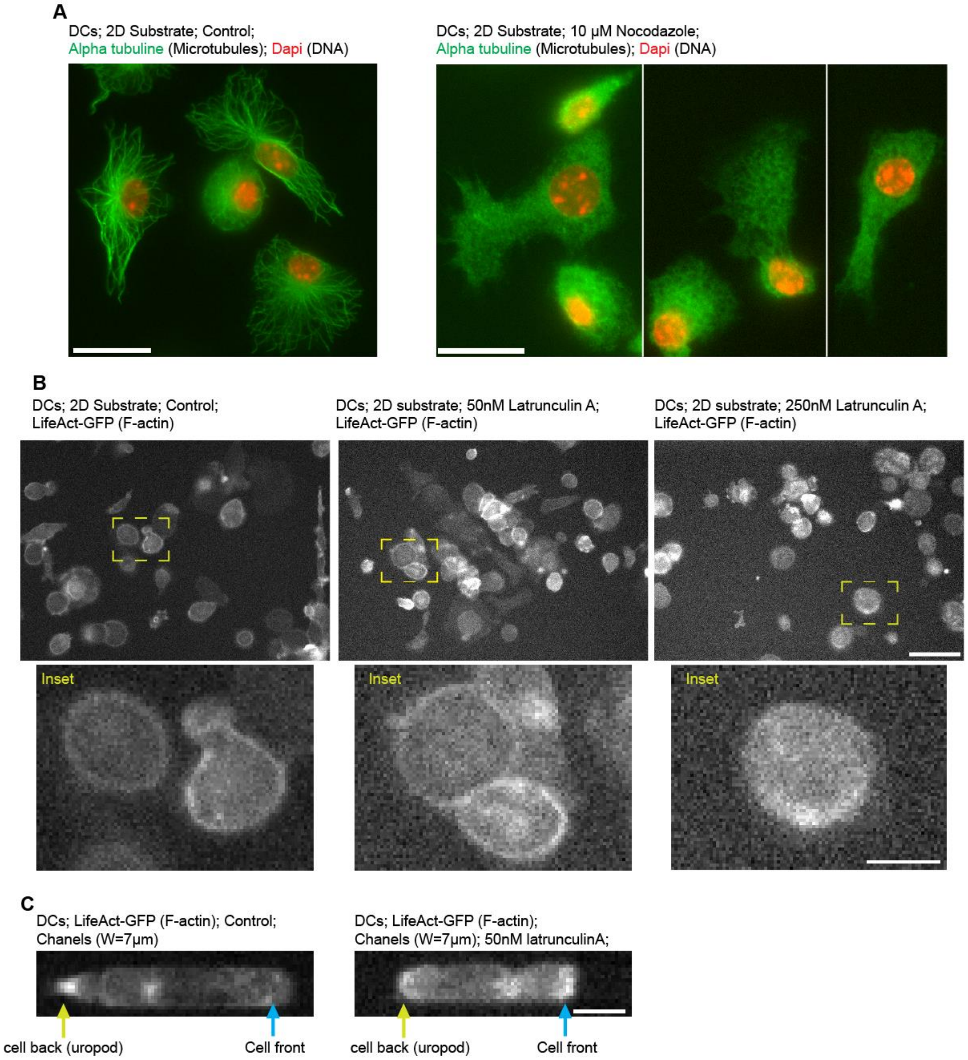

Supplementary Figure 3: The microtubule network is disrupted with 10 µM nocodazole treatment and treatment with 250 nM latrunculin A disrupts the actin cytoskeleton in dendritic cells. Representative images of immunostained DCs plated on 2D substrates for 6h after treatment with DMSO (**A**- Left)**,** 10 µM (**A**-Right) of Nocodazole, DMSO (**B**-left), 50nM (**B**-middle), 250nM (**B**-right) of Latrunculin A**.** Note the decrease in the number of polarized cells when the Latrunculin A concentration increases**.** Note that 250 nM of latrunculin A disrupts DCs cortex. (**C**) Representative image of LifeAct-GFP expressing, DMSO (**C**-Left) or 50 nM latrunculin A (**C**-right) treated DC in 7 µm channels. Yellow and Blue arrows indicate respectively the cell back and front. Scale bar (**A**)**,** (**B-**up) and (**C**) = 20 µm; (**B**)-inset = 10 µm

Supplementary Figure 4

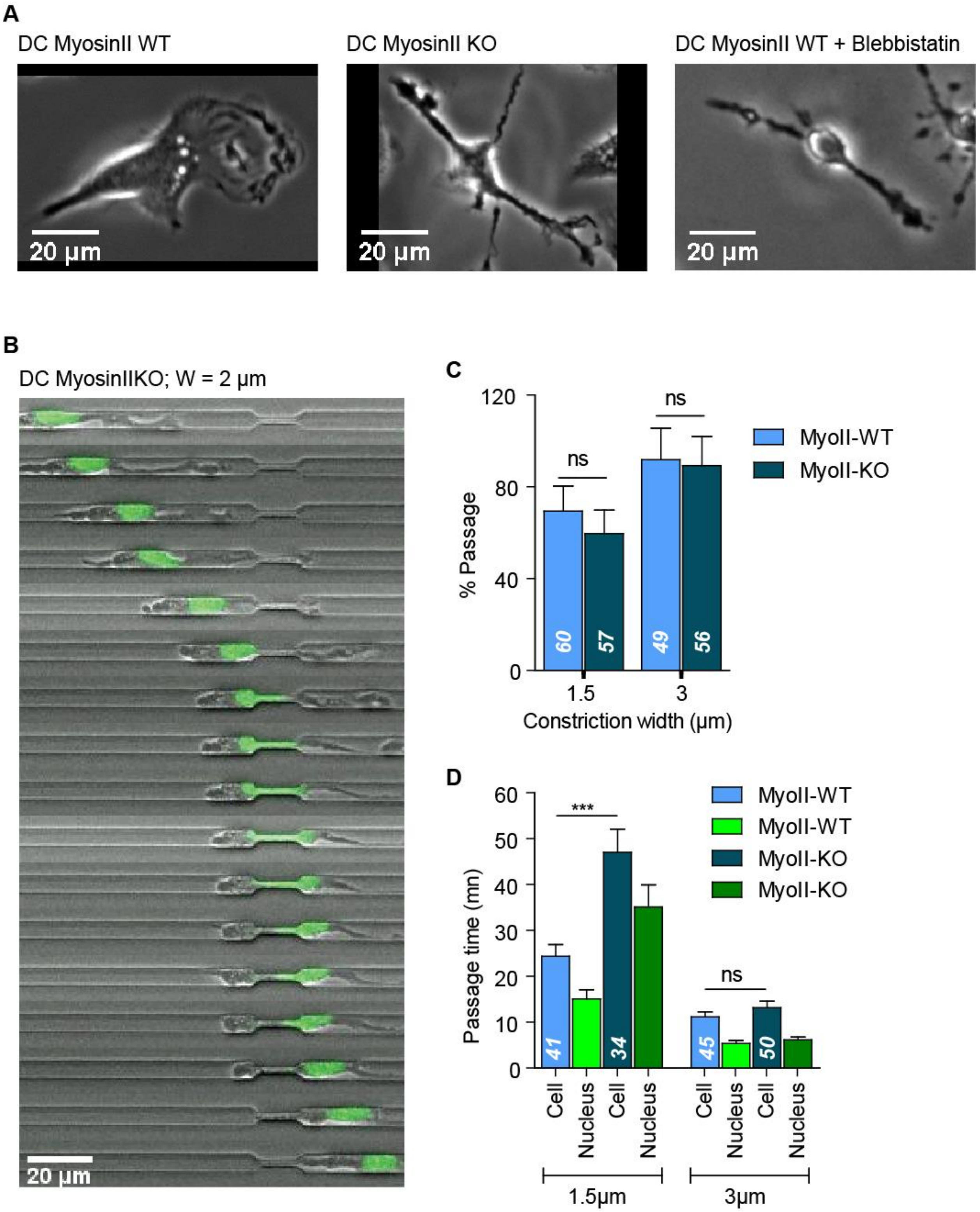

Supplementary Figure 4: Nuclear squeezing is independent of myosin II activity even in the smallest constrictions. (**A**) Representative phase contrast images of wild type, myosin IIA conditional knock out and 50 µM blebbistatin treated wild type DCs on 2D substrates. An increase of dendrites was observed upon myosin II inhibition and KO. (**B**) Montage of a myosin II KO DC migrating through a 2 µm wide constriction. Note the long cell front and cell rear; a hallmark of myosin II inhibited/KO DCs. (**C**) Percentage of passage as a function of the constriction cross section area. (**D**) Cell (blue) and nuclear (green) passage time of WT and myosin IIA KO DCs. myosin II depletion induced a significant increase in the cell as well as the nuclear passage time in 1.5 µm constrictions. Numbers in bars represent the number of cells scored. P_value: ***, ** and * indicate < 0.0001, < 0.001, and < 0.01. ns = non-significant. Statistical test: Fisher test for (**C**), Mann-Whitney test for (D). W: constriction width. Error bars in (**C**) and (**D**) represent standard deviation.

Supplementary Figure 5

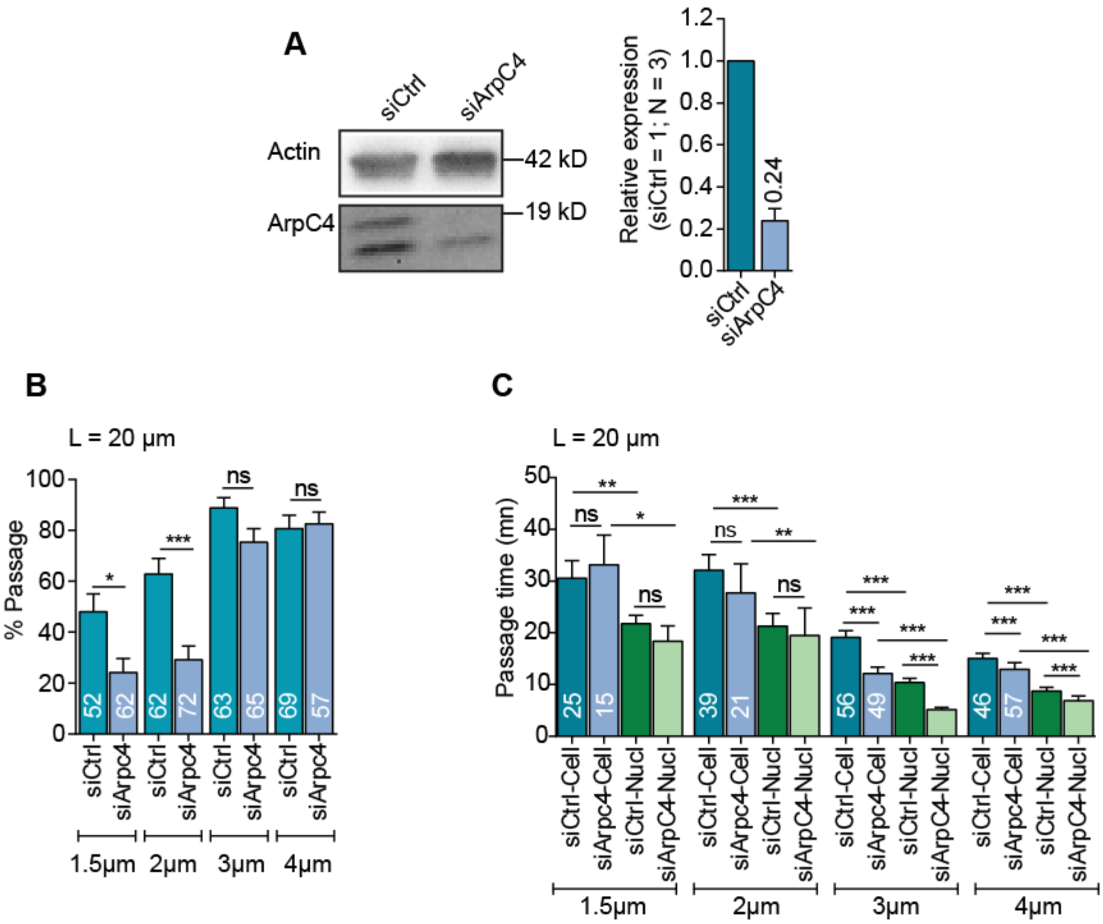

Supplementary Figure 5: Arp2/3 inhibition and depletion in DCs. (**A**) Western blot quantifying the relative expression of ArpC4 in DCs electroporated with non-targeting control or ArpC4 siRNA. (**B**) Percentage of passage, and (**C**) passage time in 1.5, 2, 3 and 4 µm wide constrictions of DCs treated as indicated. siCtrl = control siRNA; siArpC4 = ArpC4 siRNA. (**B**), (**C**): numbers represent the number of cells observed. Error bars are standard error of the mean. P_value: ***, ** and * indicate < 0.0001, < 0.001, and < 0.01. ns = non-significant. Statistical test: Fisher test for (**B**), Mann-Whitney test for (**C**). L: constriction length.

Supplementary Figure 6

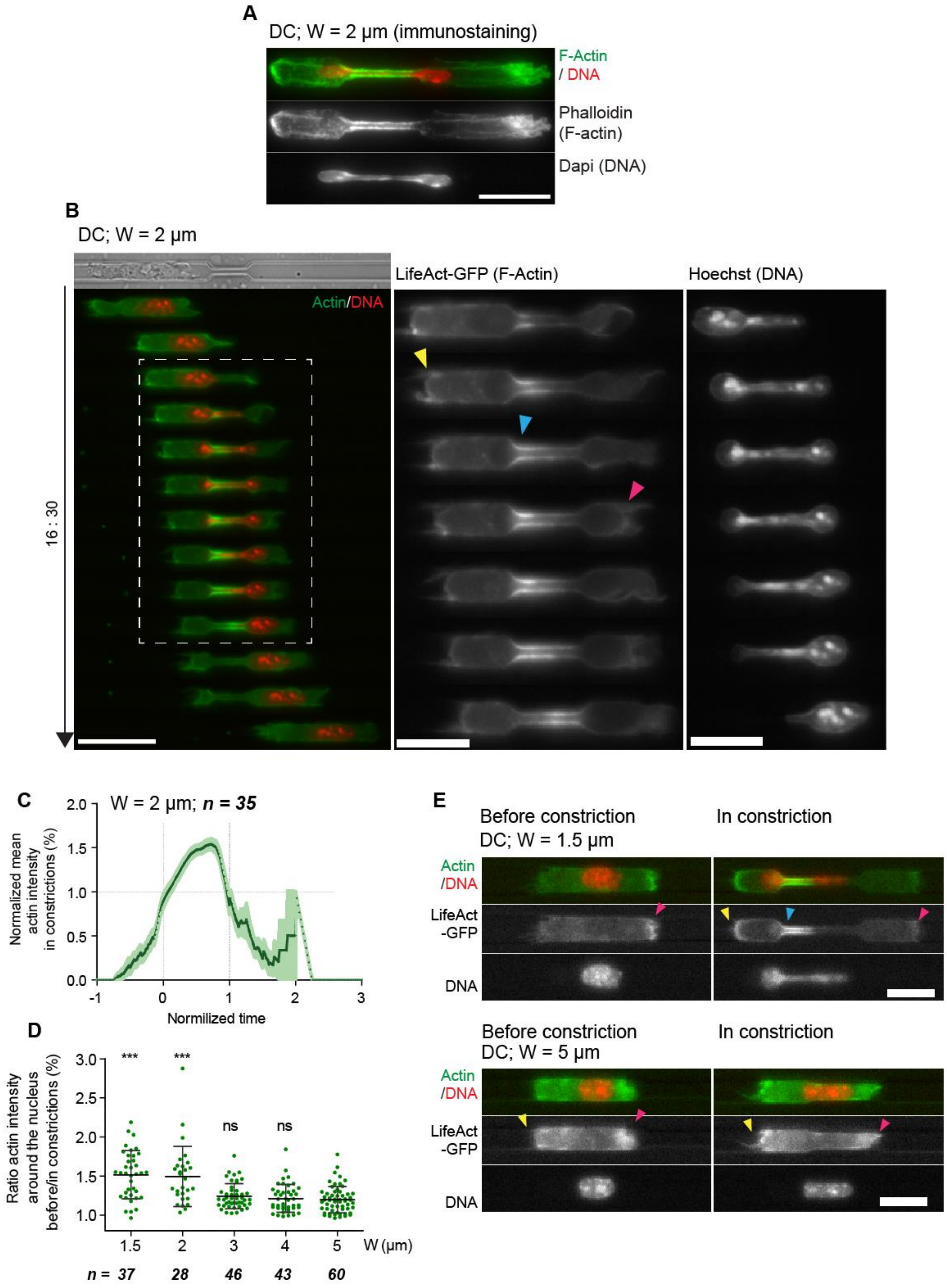

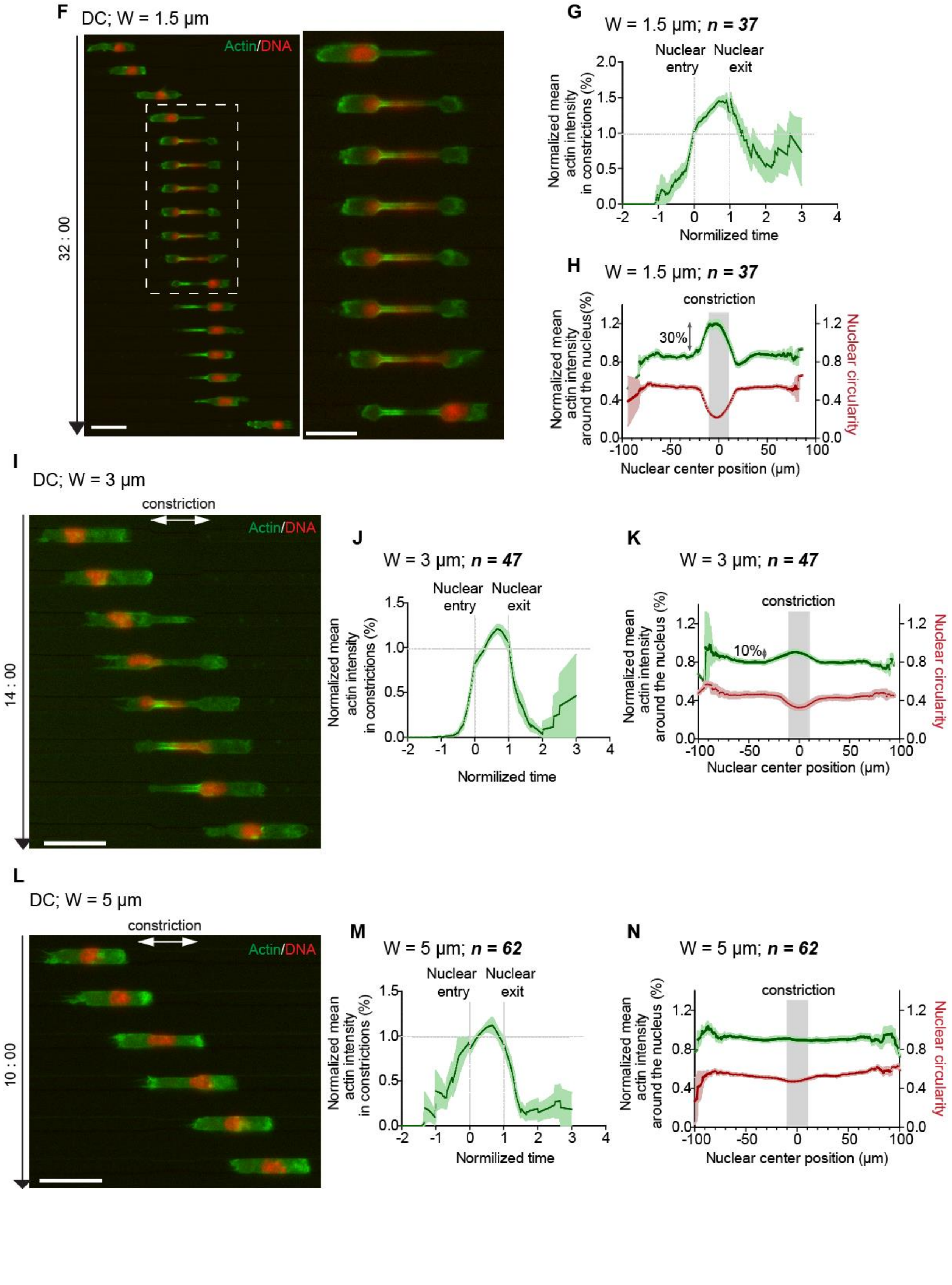
F
DC; W = 1.5 µm
Actin/DNA
32 : 00
G
W = 1.5 µm; n = 37
Nuclear entry
Nuclear exit
Normalized mean actin intensity in constrictions (%)
Normilized time
H
W = 1.5 µm; n = 37
constriction
30%
Normalized mean actin intensity around the nucleus(%)
Nuclear circularity
Nuclear center position (µm)
I
DC; W = 3 µm
constriction
Actin/DNA
14 : 00
J
W = 3 µm; n = 47
Nuclear entry
Nuclear exit
Normalized mean actin intensity in constrictions (%)
Normilized time
K
W = 3 µm; n = 47
constriction
10%
Normalized mean actin intensity around the nucleus (%)
Nuclear circularity
Nuclear center position (µm)
L
DC; W = 5 µm
constriction
Actin/DNA
10 : 00
M
W = 5 µm; n = 62
Nuclear entry
Nuclear exit
Normalized mean actin intensity in constrictions (%)
Normilized time
N
W = 5 µm; n = 62
constriction
Normalized mean actin intensity around the nucleus (%)
Nuclear circularity
Nuclear center position (µm)

Supplementary Figure 6: Accumulation of actin filaments around the nucleus occurs only for high nuclear deformation. (**A**) F-actin localization (Phalloidin staining) in DC in a 2 µm wide constriction. Scale bar: 15 µm (**B**) Sequential images of a representative DC expressing LifeAct-GFP (actin, green) and stained with Hoechst (DNA, red) migrating through a 2 µm wide constriction. Yellow, blue and pink arrows point to actin accumulation respectively at the cell rear, at the nucleus and at the cell front. Scale bar for the overlay: 30 µm; actin and DNA: 15 µm. Time in minutes: seconds. (**C**) Normalized mean actin intensity in 2 µm wide constrictions as a function of time relative to nuclear entry and exit. (**D**) Ratio of the normalized mean actin intensity inside the constriction relative to before the constriction as a function of the constriction width. Lines are for mean and bars for standard deviation. Statistical test was against the value for 5 µm wide constrictions. ***, for P_value < 0.0001. ns = non-significant. (**E**) Representative sequential images of DCs before and inside a 1.5 µm and 5 µm wide constriction. Scale bar 15 µm. (**F**), (**I**) and (**L**) Sequential images of LifeAct-GFP (actin, green) expressing DCs stained with Hoechst (DNA, red), crossing respectively a 1.5, 3 and 5 µm wide constriction. Scale bar: (**F**)-left; (**I**) and (**L**) 30 µm. (**F**)-right: 20 µm (**G**), (**J**) and (**M**) Normalized mean actin intensity as a function of the normalized time relative to nuclear entry and exit. (**H**), (**K**) and (**N**) Green: normalized mean actin intensity around the nucleus of cells passing through constrictions. Red: Nuclear circularity as a function of the nuclear position. 0 indicates the center of the constriction, grey bars represent the constriction. Dark green/red: mean; light green/red: standard error of the mean. Note the decrease in actin accumulation in constrictions, around the nucleus, as a function of constrictions widths. W: constriction width.

Supplementary Figure 7

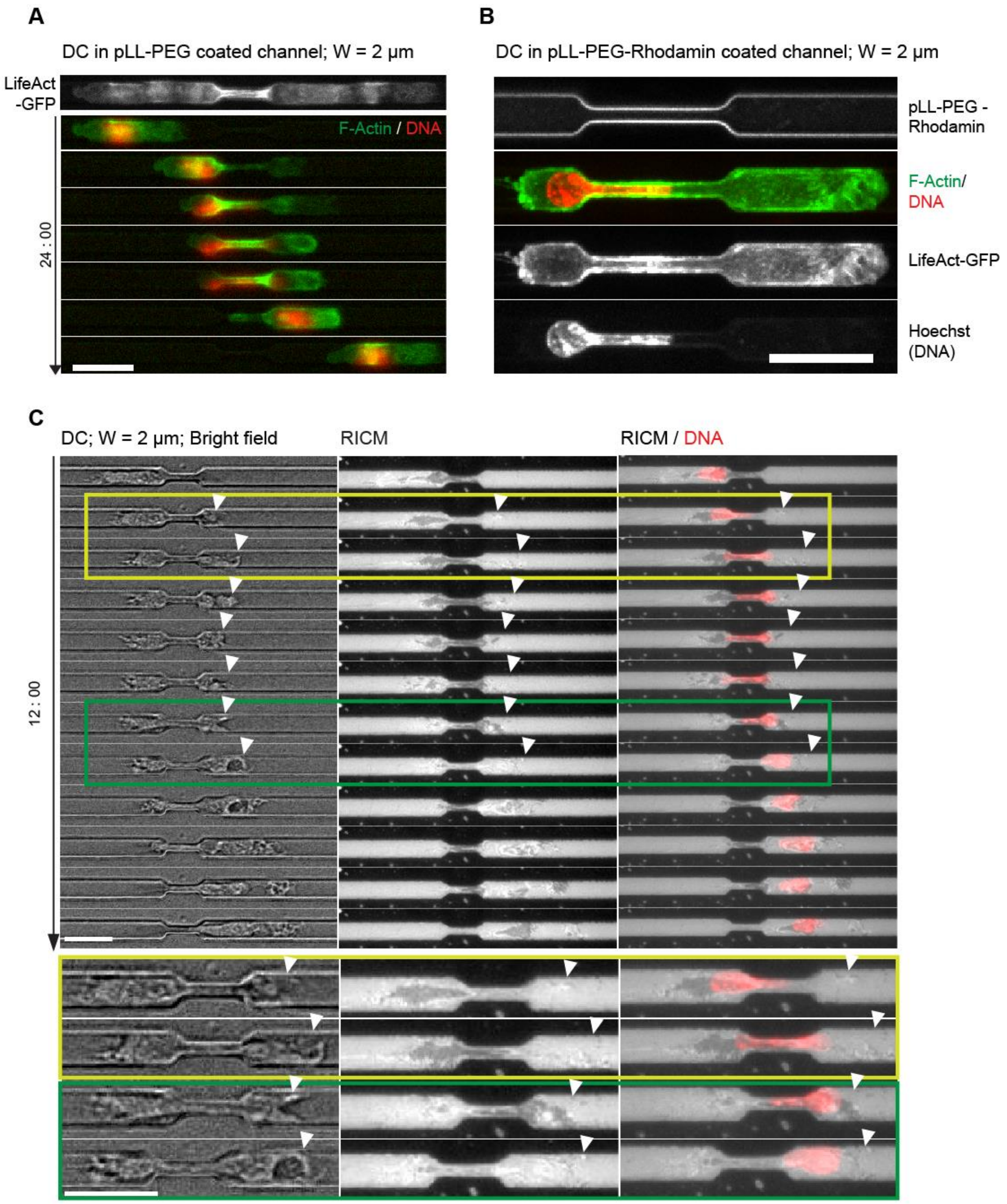

Supplementary Figure 7: Actin accumulation during nuclear passage through constrictions does not rely on adhesion complexes and no contact between the cell and the substrate is observed at the cell front during nuclear passage. (**A**) and (**B**) Representative image of DCs expressing LifeAct-GFP (actin, green) stained with Hoechst (DNA, red) migrating through pLL-PEG coated constrictions. (**A**)-Top: Maximun projection of the 13 time points of a timelapse movie. (**A**)-Bottom: Sequential images of a representative cell crossing a constriction. Time in minutes: seconds. (**B**) Maximun projection of a Z-stack acquisition. (**C**) Sequential images of a representative IiKO iDC in a 2 µm wide constriction coated with pLL-PEG. Left: Bright field images to visualize the cell body. Middle: Reflection Interference Contrast Microscopy (RICM) to visualize cell-substrate contact areas (in dark grey). Right: Overlay of the RICM signal with the Hoechst signal for DNA localization (red). Arrows indicates cell front protrusion. During nuclear passage, the front was not in close contact with the bottom substrate (8 over 8 observed cells). Scale Bar: 20 µm. W: constriction width.

Supplementary Figure 8

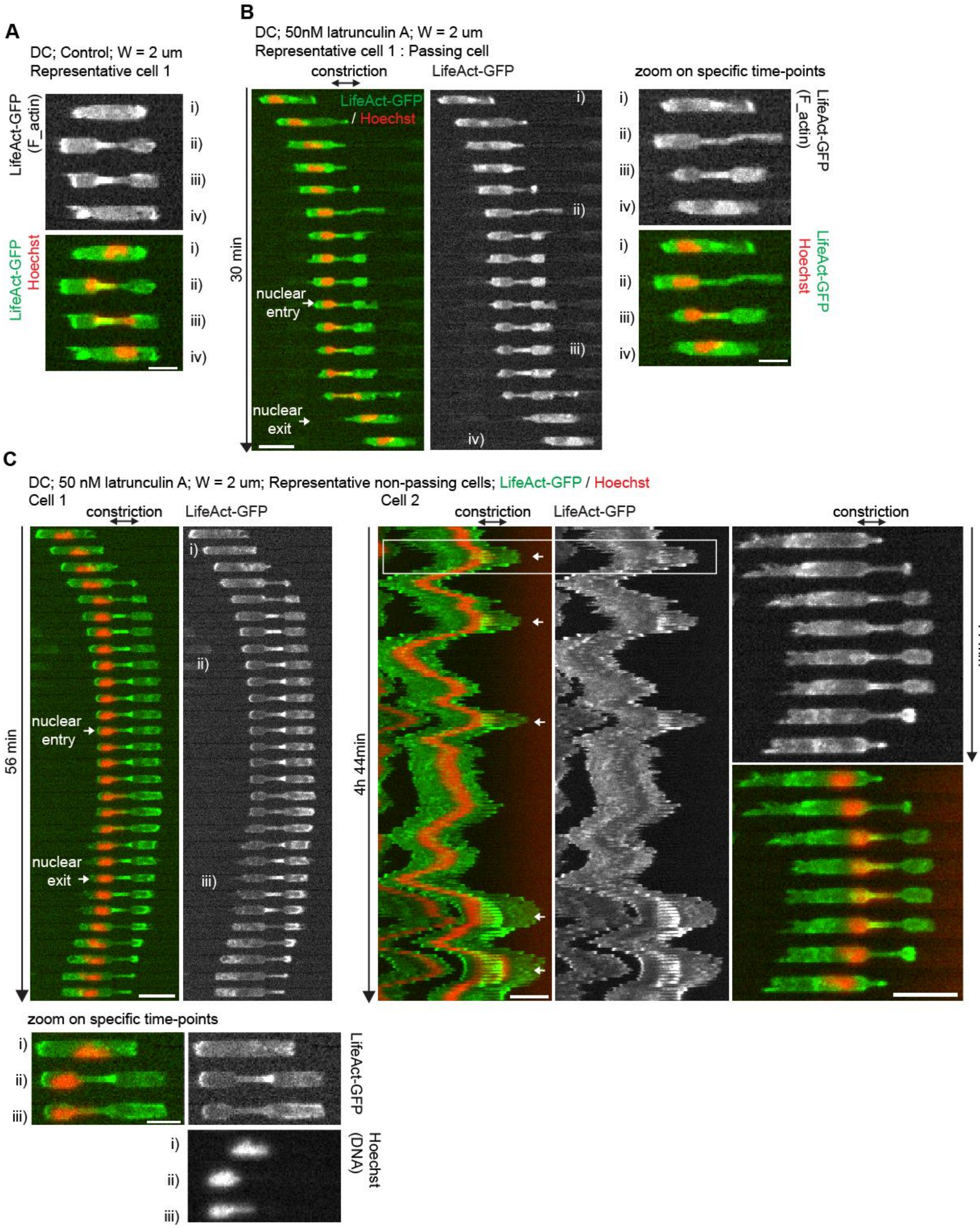

Supplementary Figure 8: Low doses of latrunculin A (50nM) perturbs the perinuclear actin network formation in constrictions (**A**) Sequential images of a representative LifeAct-GFP expressing (actin, green) DC stained with Hoechst (DNA, red) treated with DMSO passing a 2 µm wide constriction. One can observe the actin increase in constriction upon nuclear entry. (**B**) Sequential images of a representative LifeAct-GFP expressing (actin, green) DC stained with Hoechst (DNA, red) treated with 50 nM of latrunculin A passing a 2 µm wide constriction. One can observe that actin does not increase in constriciton upon nuclear entry (**C**) Sequential images of 2 representative LifeAct-GFP expressing (actin, green) DCs stained with Hoechst (DNA, red) and treated with 50nM of Latrunculin A attempting to pass a 2 µm wide constriction. Representative cell 1: actin starts accumulating in constriction prior nuclear entry and no actin accumulation is observed around the nucleus when it enters the constriction. Representative cell 2 fails to pass the constriction after 5 trials. One can observe the increase of actin in constriction at each trial. Scale bar: 30 µm. L: constrictions length. W: constriction width. i): time point before cell and nuclear entry in constriction; ii): time point after cell but before nuclear entry in constriction; iii): time point after cell and nuclear entry in constriction. iii): time point after cell exit from the constriction.

Supplementary Figure 9

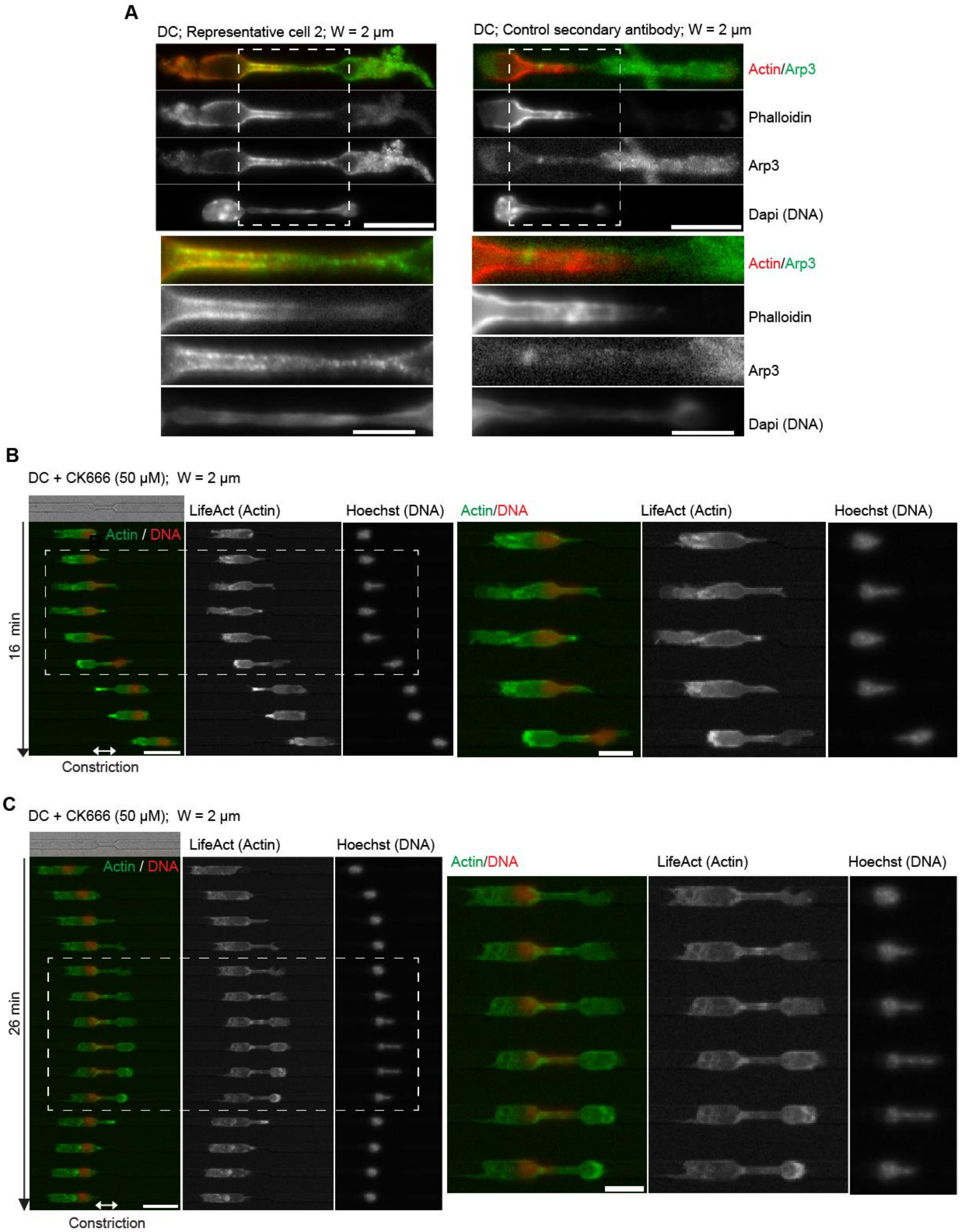

Supplementary Figure 9: The F-actin meshwork formed around the nucleus is nucleated by Arp2/3. (**A**) Immunostaining of the Arp2/3 complex localization in DCs passing 2 µm wide constrictions. F-actin was stained with phalloidin. Control secondary antibody staining (right) shows that the accumulation of Arp3 staining co-localized with F-actin was specific. Bottom: Magnification of the image at the constriction for a better visualization of the Arp3/F-actin co-localization around the nucleus. Scale bar top: 15 µm; bottom: 5 µm. (**B**) Sequential images of a representative LifeAct-GFP expressing (actin, green) DC stained with Hoechst (DNA, red) treated with 50µM of the Arp2/3 inhibitor CK666 but still passing a constriction. No actin increase was observed around the nucleus during its deformation through the constriction. (**C**) Sequential images of a representative LifeAct-GFP (actin, green) expressing DC stained with Hoechst (DNA, red) treated with 50 µM of the Arp2/3 inhibitor CK666, and failing to pass a constriction. A transient and faint actin increase in the constriction correlating with nuclear entry could be observed. Scale bar (**B**) and (**C**) entire field: 30 µm; Zoom: 15 µm. min for minutes. W: constriction width. L: constriction length.

Supplementary Figure 10

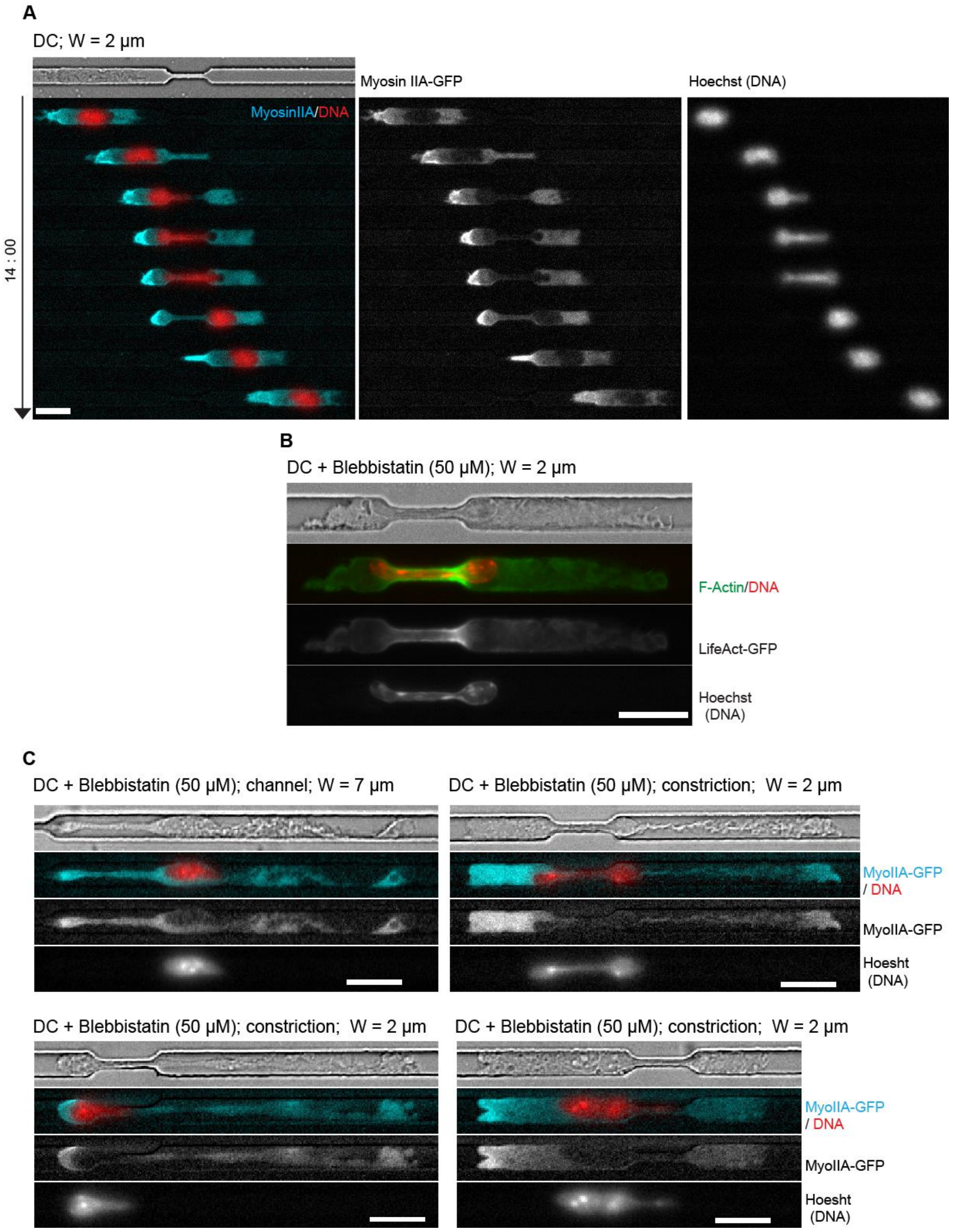

Supplementary Figure 10: The F-actin accumulation around the nucleus does not co-localize with myosin II and does not depend on myosin II activity. (**A**) Sequential images of a representative myosin IIA-GFP (cyan) expressing DC migrating through a 2 µm wide constriction. myosin IIA-GFP accumulated at the cell back but not around the nucleus inside the constriction. Time in minutes: seconds. (**B**) Representative image of 19 over 21 cells observed for LifeAct-GFP (green) expressing DC stained with Hoechst (DNA, red) and treated with 50 µM blebbistatin, migrating in a 2 µm wide constriction. A single time point was acquired to avoid damaging blebbistatin with the excitation light. The long cell front was typical of myosin II inhibited DCs. Scale bar 15 µm. (**C**) Representative images of myosin IIA-GFP (cyan) DCs stained with Hoechst (DNA, red) and treated with 50 µM blebbistatin. Single time points were acquired to avoid damaging blebbistatin with the excitation light. Top-left: the cell just exited the constriction; top-right: the cell and its nucleus were fully engaged in the constriction; bottom: the cells were fully engaged but the nucleus was just starting to enter the constriction. The absence of myosin IIA-GFP accumulation at the cell back demonstrated efficient inhibition of myosin II motor activity by blebbistatin inside the channels, and the long cell front extension was characteristic of blebbistatin treated and myosin IIA KO DCs. W: constriction width.

Supplementary Figure 11

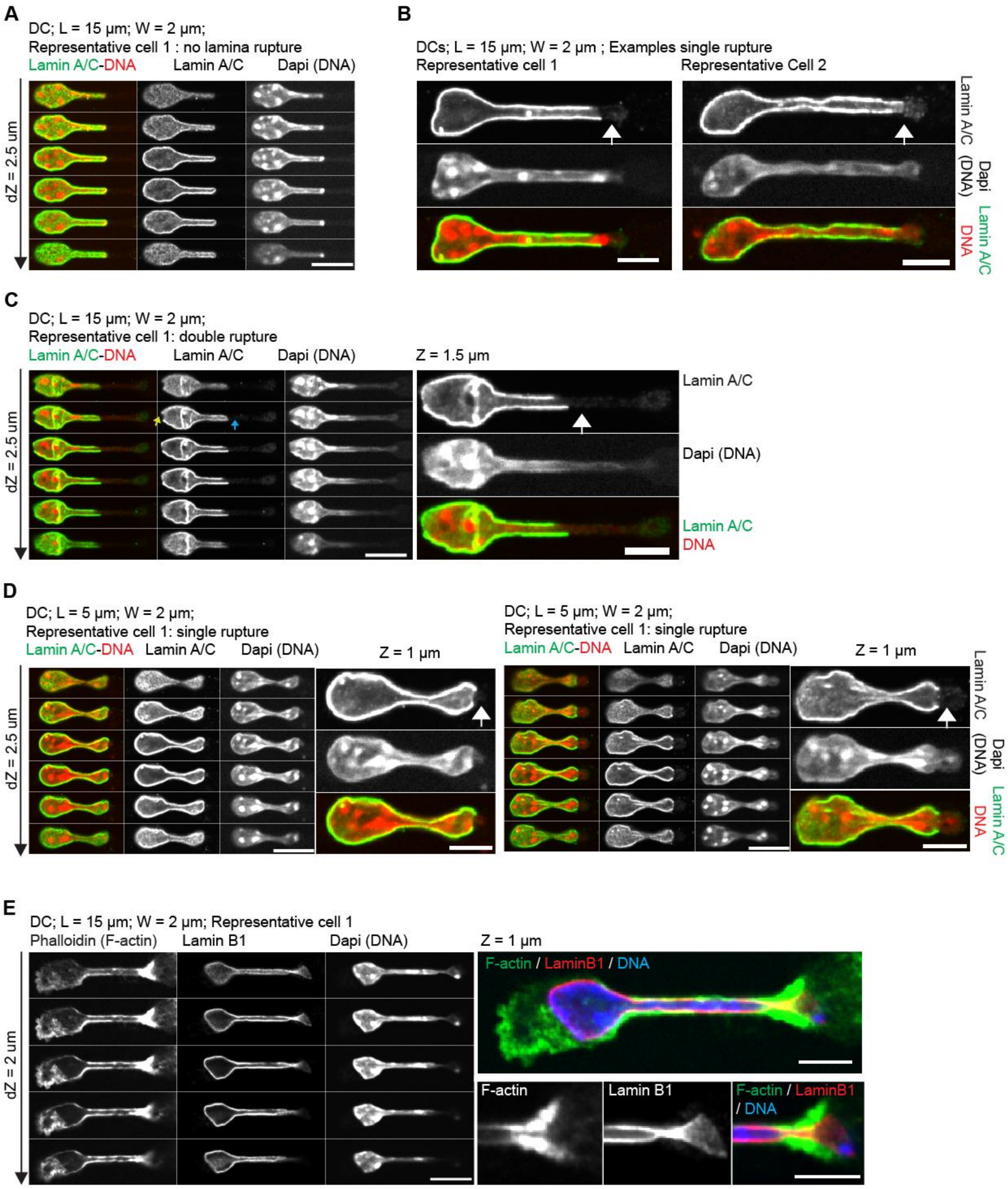

Supplementary Figure 11: Nuclear deformation through narrow constrictions leads to lamina breakage. Representative images of immunostained DCs in 2 µm wide and 15 µm (**A**), (**B**), (**C**), (**E**), or 5 µm (**D**) long constrictions. White arrows to show ruptured lamina network, yellow and blue arrows show lamina rupture respectively at the back and front of the nucleus in (**C**). Scale bar: 10 µm (**A**, **C**-left, non-zoomed images in **D**, **E**-left); 5 µm (**B**, **C**-right, Zoom in **D**, **E**-right).

Supplementary Figure 12

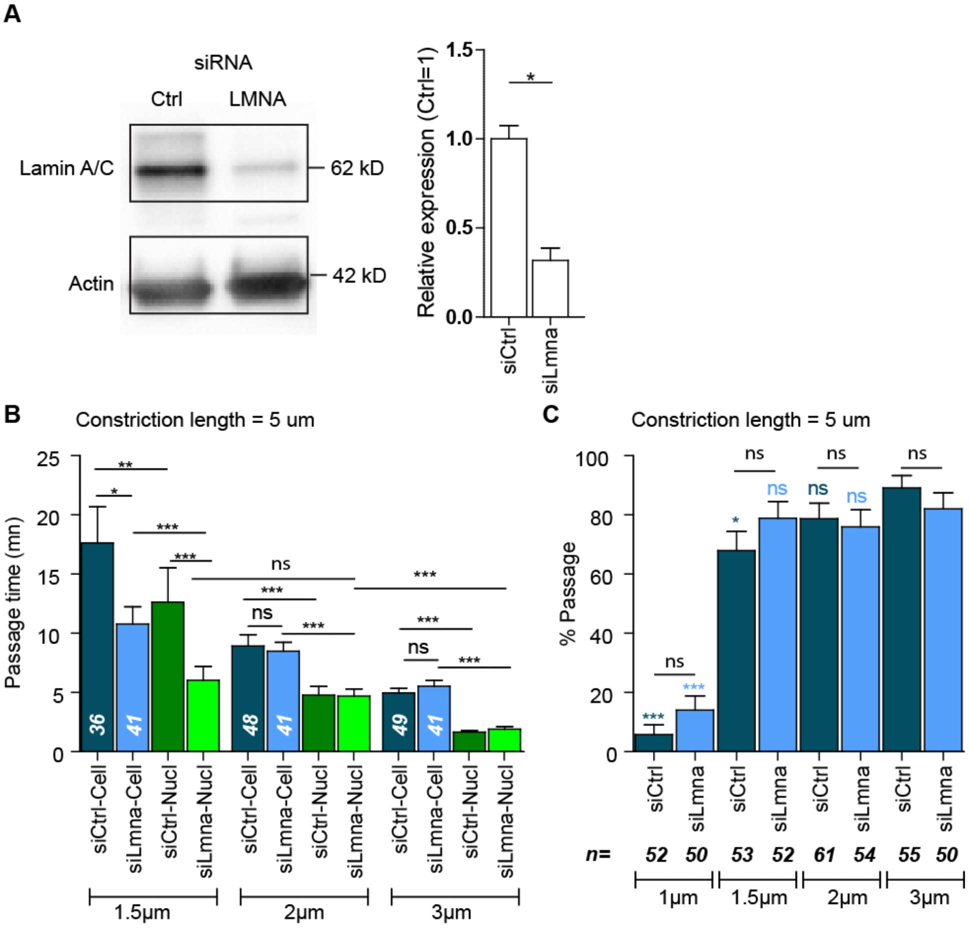

Supplementary Figure 12: Effect of lamin A/C depletion on DCs passage through constrictions. (**A**) Quantification of lamin A/C depletion after siRNA treatment in DCs. G-actin was used as a loading control. The Western blot on the left shows a typical experiment, while the quantification on the right shows mean and standard error for 4 independent experiments. (**B**) Cell (blue) and nuclear (green) passage time for control and lamin A/C depleted DCs as a function of constriction widths. Constrictions were 5 µm long. Numbers in bars represent the total number of cells scored. (**C**) Percentage of passage of control and Lamin A/C depleted DCs through 5 µm long constrictions. The percentage of passage in 1 µm constrictions was significantly different from zero for Lamin A/C depleted cells (P value = 0.02046) but not for control cells (P value=0.3134). n represents the number of cells. Unless when indicated with a spanner, statistical test was against the value for 5 µm wide constrictions. Error bars represent standard deviation**.**

Supplementary Figure 13

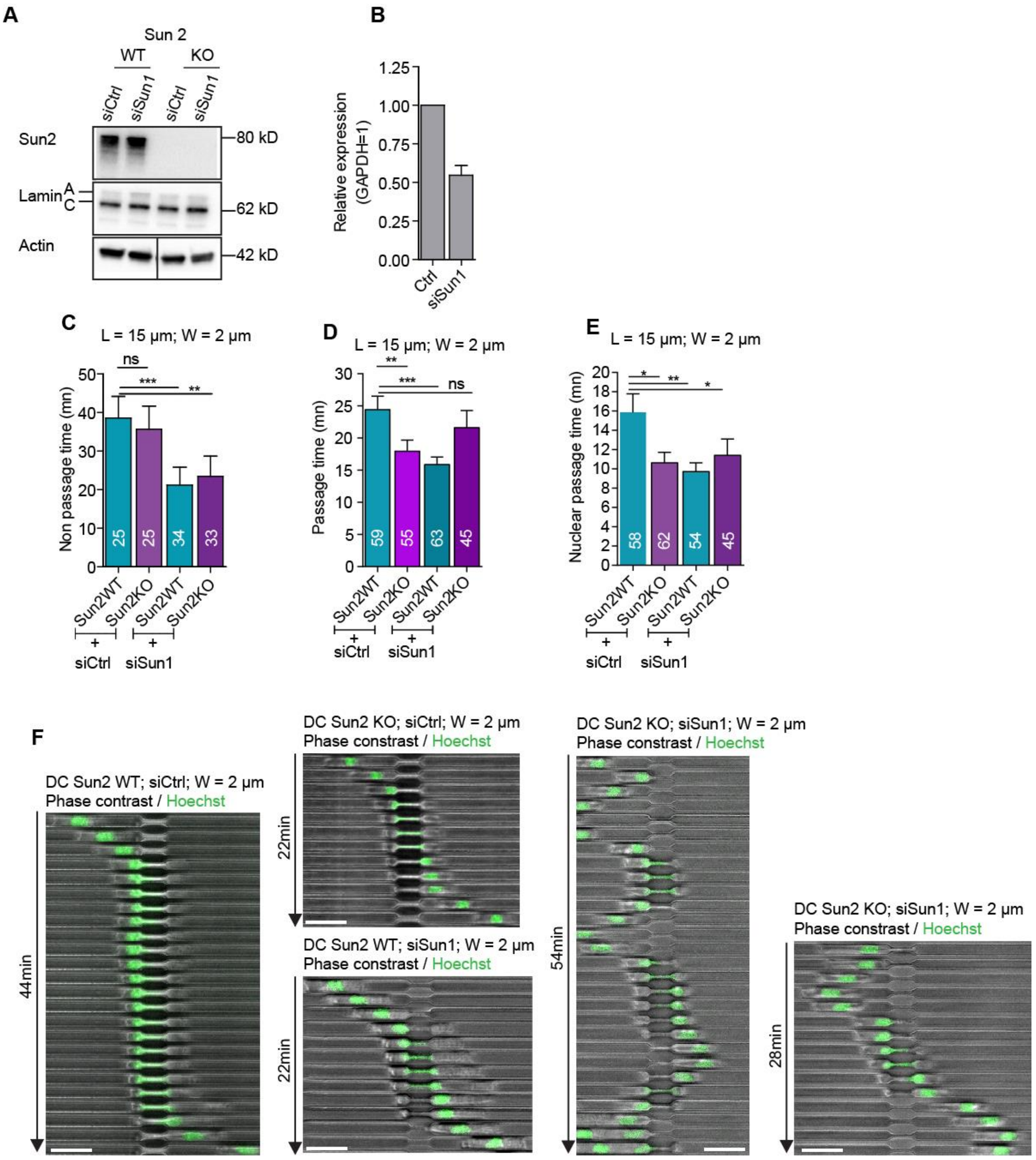

Supplementary Figure 13: Sun 1 and Sun 2 depletion impact cell persistence but not the efficiency of passage though micrometric constrictions nor the perinuclear actin formation. (A) Representative western blot of Sun 2 WT and KO dendritic cells electroporated with non-targeting siRNA. One can observe the depletion of Sun 2 without an effect on lamin A/C expression level. (**B**) qPCR based quantification of sun1 depletion after DCs electroporation with non-targeting or sun1 siRNA. (**C**) Non passage time, (**D**) cell passage time and (**E**) Nuclear passage time in DCs treated as indicated. siCtrl = control siRNA; siSun1 = Sun1 siRNA. Numbers represent the number of cells observed. Error bars are standard error of the mean. P_value: ** and * indicate < 0.001, and < 0.01. Statistical test: Mann-Whitney test. (**F**) Sequential images of a representative DCs treated as indicated in 2 µm wide constrictions. Note the frequent change in direction observed in the Sun2 KO DCs electroporated with the sun 1 siRNA. Scale bar: 30 µm.

Supplementary Figure 14

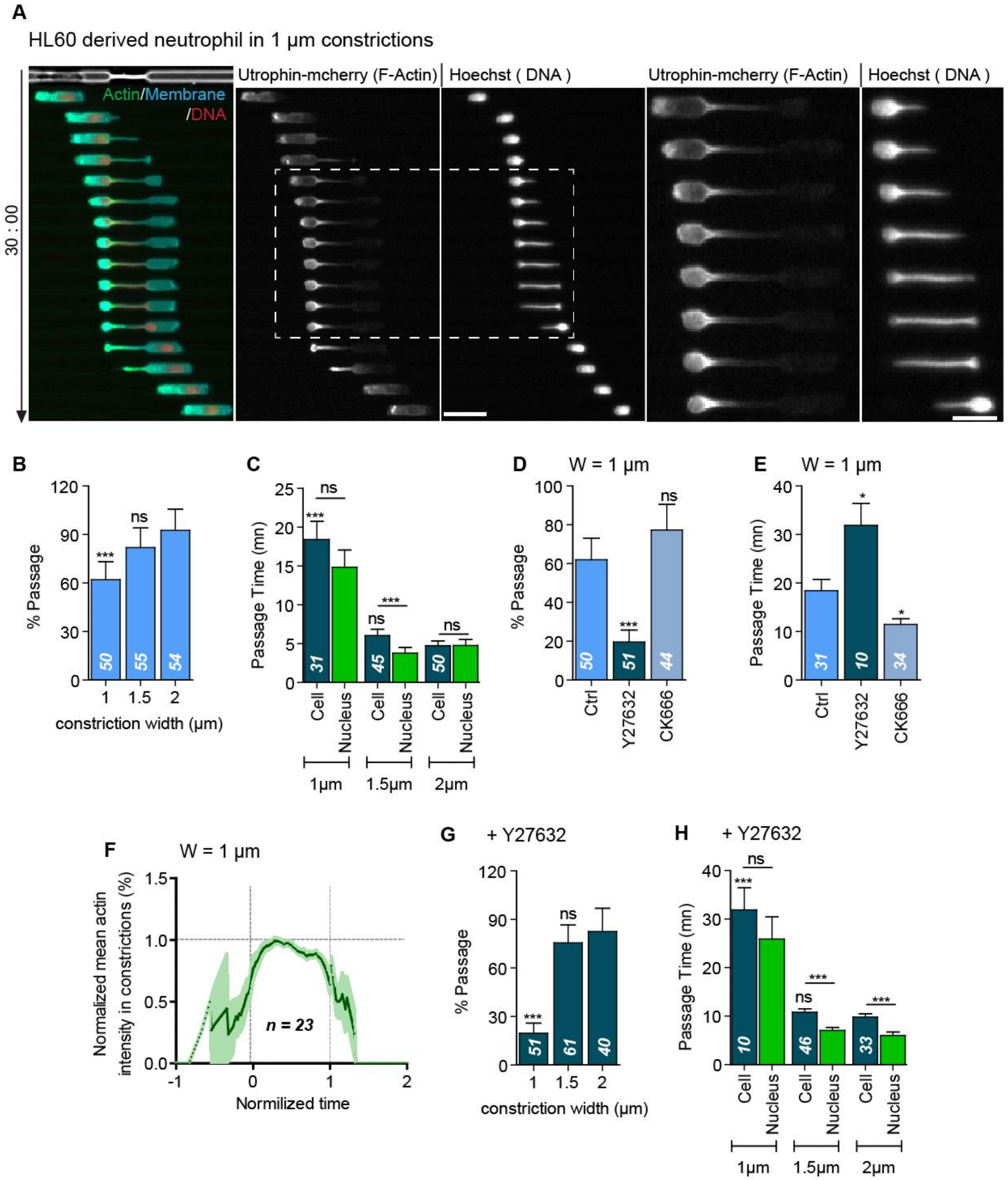

Supplementary Figure 14: Neutrophil passage through 1.5 µm wide constrictions does not require Arp2/3 or contractility, but passage through 1 µm requires

contractility and not Arp2/3. (**A**) Sequential images of a representative HL60-derived neutrophil migrating through a 20 µm long, 1 µm wide constriction. The plasma membrane (blue) was visualized with Lyn-Emerald expression, the F-actin cytoskeleton (green) with Utrophin-mCherry and DNA with Hoechst (red). A faint increase of F-actin could be observed in the constriction during nuclear passage, but most of the F-actin was located at the cell rear. Zoom on the nuclear passage event (two most right panels) illustrates nuclear deformation in the 1 µm wide constriction. Scale bar 30 µm; zoomed image, 15 µm. Time in minutes: seconds. (**B**) Percentage of passage of control HL60-derived neutrophils, as a function of the constrictions dimensions. More than 60% of control cells could still cross the 1 µm wide constrictions which DCs cannot cross. (**C**) Passage time of control HL60-derived neutrophils as a function of the constrictions dimensions. (**D**) and (**E**) Respectively percentage of passage and passage time for 1 µm wide constrictions, after ROCK (Y27632) or Arp2/3 (CK666) inhibition. (**F**) Mean actin intensity inside 1 µm constrictions, normalized by the mean actin intensity in the whole cell. No actin enrichment was observed inside constrictions. (**G**) and (**H**) Respectively percentage of passage and passage time as a function of constrictions dimension for ROCK inhibited HL60-derived neutrophils (Y27632 treatment). ROCK inhibition did not affect HL60-derived neutrophils passage through constrictions larger than 1 µm. (**B**), (**C**), (**G**) and (**H**) Unless when indicated by a spanner, statistical test was against the value for 2 µm wide constrictions. (**D**) and (**E**) Statistical tests performed against Ctrl (for control). Quantification was done with actin-GFP expressing HL60 derived neutrophils in 5 µm long constrictions. P_values: ***, * indicate < 0.0001, < 0.01. ns = non-significant. Numbers in bars represent the number of cells observed. W: constriction width. Error bars represent standard deviation.

Supplementary Figure 15

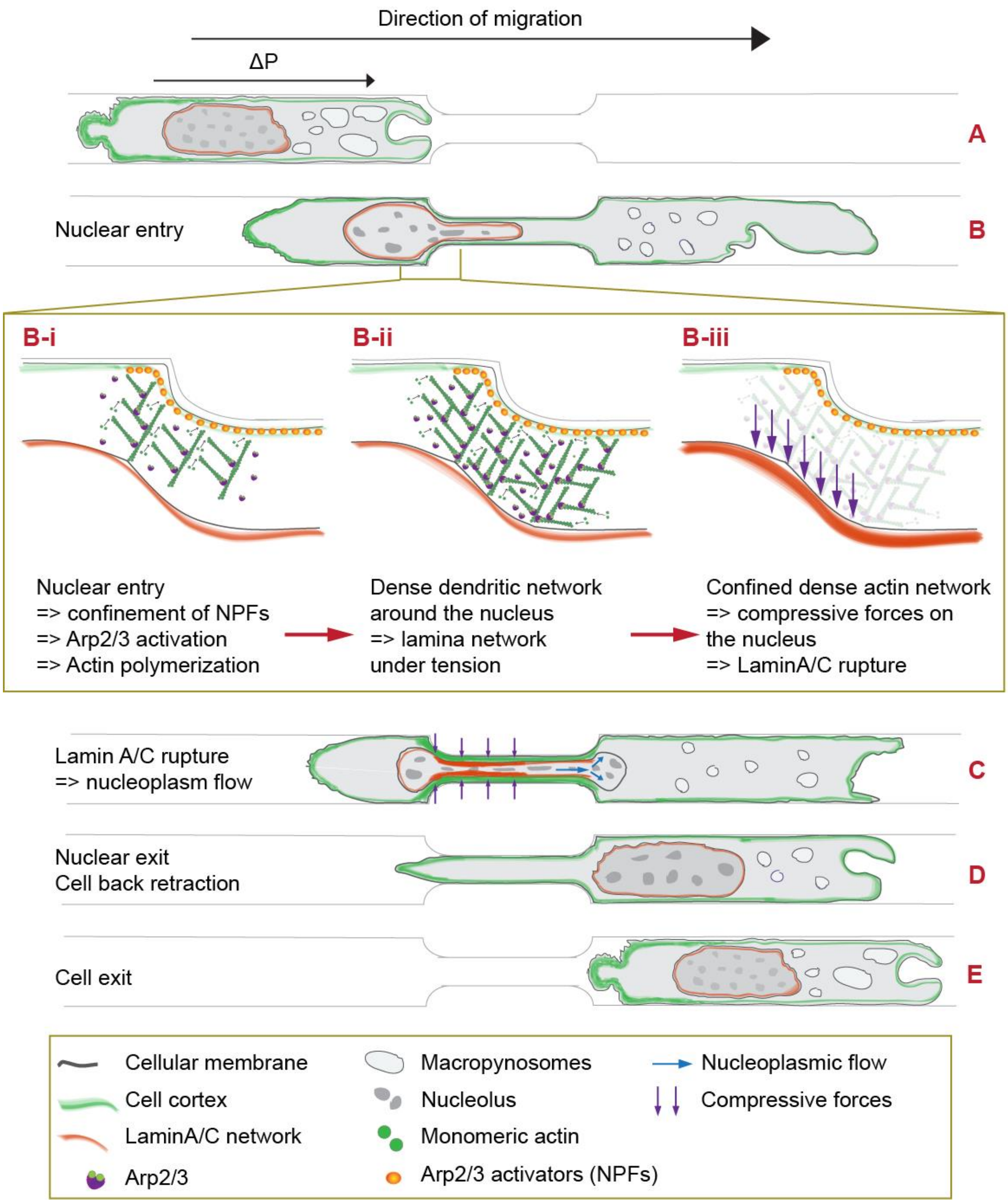

Supplementary Figure 15: Working model for nuclear passage through micrometric constrictions. A) Cell in channel with a front-back pressure gradient ΔP. Black arrows illustrate the direction of the internal pressure gradient. **B**) Cell entering a

constriction. ΔP allows first nuclear protrusion which confinement induces a local confinement of the plasma membrane, the nuclear membrane and the lamina. **B-i**)) Confinement of Arp2/3 activators such as Wave 2 thus to local Arp2/3 activation. **B-ii**)) The positive feedback loop between confinement and Wave2 activation generates the dense actin meshwork. **B-iii**)) The confined dendritic network produce compressive forces (purple arrows) applied on the nucleus. The nuclear membrane reacts as a fluid and flow while the viscoelastic lamina network ruptures. **C**) Lamin A/C rupture releases the nuclear surface tension allowing the nucleoplasm which is a viscous liquid at this scale to flow in the constriction. The lamina network will repolymerize at the constriction exit. **D**) The internal pressure gradient drives back retraction. **E**) The cell exits the constriction and goes back to its initial state (**A**).

Supplementary Table 1: Dimensions of constrictions used in the study. L = length; W = width; H = height; C.S.A. = cross-section area; SD = standard deviation. SD C.S.A. is estimated as the product between W and SD of the height (SD H).

L (µm)	W (µm)	Mean H (µm)	SD H (µm)	Mean C.S.A. (µm²)	SD C.S.A. (µm²)
**20/15**	1	2.26	0.23	2.26	0.23
	1.5	2.96	0.28	4.44	0.42
	2	3.43	0.33	6.86	0.66
	3	4.2	0.11	12.6	0.33
	4	4.39	0.1	17.56	0.4
	5	4.56	0.07	22.8	0.35
	7	4.7	0.11	32.9	0.77
**5**	1	2.16	0.21	2.16	0.21
	1.5	3.56	0.2	5.34	0.3
	2	3.88	0.38	7.76	0.76
	3	3.92	0.13	11.76	0.39
	4	4.24	0.12	16.96	0.48
	5	4.5	0.2	22.5	1
	7	4.81	0.12	33.67	0.84

Supplementary Table 2: Mean, standard deviation/ error on the mean, P values

Figure Panel	Condition	Mean	SEM	n	Test	P value against	P value
1E	1 µm	4	0.565686	50	Fisher test	5 µm	< 2.2e-16
	1.5 µm	40.74074	5.544113	54	Fisher test	5 µm	1.59E-07
	2 µm	61.29032	7.783879	62	Fisher test	5 µm	0.001179
	3 µm	78.68852	10.07503	61	Fisher test	5 µm	0.2407
	4 µm	82.35294	11.53172	51	Fisher test	5 µm	0.4619
	5 µm	90.56604	12.4402	53	Fisher test		
1G	1.5 µm-cell	22.41	3.485	22	Mann whitney	5 µm-cell	< 0.0001
	1.5 µm-Nucleus	15	3.076	22	Mann whitney	1.5 µm-cell	0.0179
	2 µm-cell	18.45	1.559	38	Mann whitney	5 µm-cell	< 0.0001
	2 µm-Nucleus	12.21	1.403	38	Mann whitney	2 µm-cell	< 0.0001
	3 µm-cell	10.69	0.9817	48	Mann whitney	5 µm-cell	0.6746
	3 µm-Nucleus	5.917	0.7262	48	Mann whitney	3 µm-cell	< 0.0001
	4 µm-cell	8.548	0.6468	42	Mann whitney	5 µm-cell	0.1818
	4 µm-Nucleus	4.405	0.399	42	Mann whitney	4 µm-cell	< 0.0001
	5 µm-cell	9.458	0.6036	48			
	5 µm-Nucleus	4.75	0.4279	48	Mann whitne	5 µm-cell	< 0.0001

					y		
1H	1 µm-cell	0.8515	0.1886	39			
	1 µm-Nucleus	0.3924	0.2318	37	Mann whitney	1 µm-cell	< 0.0001
	1.5 µm-cell	0.9095	0.05945	32			
	1.5 µm-Nucleus	0.599	0.06113	32	Mann whitney	1 µm-cell	0.0003
	2 µm-cell	0.7267	0.07443	26			
	2 µm-Nucleus	0.4818	0.06487	26	Mann whitney	1 µm-cell	0.0052
	3 µm-cell	0.9241	0.03128	43			
	3 µm-Nucleus	0.9528	0.05255	43	Mann whitney	1 µm-cell	0.5863
	4 µm-cell	0.9883	0.04122	36			
	4 µm-Nucleus	0.9103	0.06128	36	Mann whitney	1 µm-cell	0.3363
	5 µm-cell	0.9929	0.03656	55			
	5 µm-Nucleus	0.9907	0.04256	55	Mann whitney	1 µm-cell	0.9887
2A	DMSO	67.56757	5.441806	74			
	Blebbi	50.63291	5.624989	79	Fisher test	DMSO	0.109
	MyoIIWT	56.71642	4.280195	134			
	MyoIIKO	57.63547	4.010493	203	Fisher test	MyoIIWT	0.9888
	mDia1WT	66.66666	5.939139	63			
	mDia1KO	56.16438	5.807412	73	Fisher test	mDia1WT	0.4432
	DMSO	78.26087	9.722034	23			
	LatA_50nM	22.91667	12.6724	48	Fisher	DMSO	4.76E-05

			2		test		
	DMSO	60	8.164966	60			
	CK666	30.92784	8.438508	97	Fisher test	DMSO	0.001777
	siCtrl	71.64179	6.505819	67			
	siArpC4	30.68182	8.875284	88	Fisher test	siCtrl	2.21E-06
	Hem1WT	78.26087	6.081553	46			
	Hem1KO	28.57143	6.453628	49	Fisher test	Hem1WT	6.04E-06
	Hem1WT+ Blebbi	64.15094	6.587224	53			
	Hem1KO+Blebbi	20	5.163978	60	Fisher test	Hem1WT+ Blebbi	9.86E-06
	DMSO	68.33334	6.005399	60			
	Noco	85.96491	4.600772	57	Fisher test	DMSO	0.07748
2B	DMSO	10.3	0.9985	50			
	Blebbi	28.63	3.432	40	Mann Whitney	DMSO	< 0.0001
	MyoIIWT	13.22	1.622	76			
	MyoIIKO	19.69	1.77	125	Mann Whitney	MyoIIWT	< 0.0001
	DMSO	19.11	2.582	18			
	LatA_50nM	66.55	15.56	11	Mann Whitney	DMSO	0.0007
	DMSO	19.43	3.79	37			
	CK666	15.93	3.142	29	Mann Whitney	DMSO	0.1876
	DMSO	14.15	0.915	41			
	Noco	9.673	0.3658	49	Mann Whitney	DMSO	< 0.0001

2C	MyoIIWT	4.871	0.0851	1067			
	MyoIIKO	2.738	0.05314	2529	Mann Whitney	MyoIIWT	< 0.0001
	mDia1WT	4.431	0.0485	2692			
	mDia1KO	4.185	0.07956	1263	Mann Whitney	mDia1WT	
	DMSO	4.624	0.3926	175			
	LatA_50nM	5.529	0.2405	245	Mann Whitney	DMSO	< 0.0001
	siCtrl	3.806	0.07374	2380			
	siArpC4	5.035	0.1278	717	Mann Whitney	siCtrl	< 0.0001
2D	DMSO	20.92	4.018	24			
	Blebbi	21.26	2.863	39	Mann Whitney	DMSO	0.5705
	MyoIIWT	17.22	1.48	58			
	MyoIIKO	41.6	7.509	76	Mann Whitney	MyoIIWT	0.0002
	DMSO	12.8	61.12	5			
	LatA_50nM	3.929	20.47	33	Mann Whitney	DMSO	0.0015
	DMSO	36.7	8.421	23			
	CK666	163.4	23.12	67	Mann Whitney	DMSO	0.0003
	DMSO	15.63	1.671	19			
	Noco	10	0.8452	8	Mann Whitney	DMSO	0.0497
2E	Control	45	11.1243	20			
	LatA_50nM	40.54054	8.071503	37	Fisher test	Control	0.9665
	CK666	44.26229	8.51828	61	Fisher	Control	1

			3		test		
4A	Control non passage	29.16667	11.02396	24			
	Control passage	100	16.66667	34	Fisher test	Control non passage	4.35E-09
	CK666 non passage	25.37313	6.153889	67			
	CK666 passage	40	11.54701	30	Fisher test	CK666 non passage	0.3383
	CK666 passage				Fisher test	Control passage	6.77E-08
5D	W = 2 µm; L=15 µm	86.7347	3.426431	98			
	W = 2 µm; L=5 µm	43.24324	8.144549	37	Fischer test	W = 2 µm; L=15 µm	3.31E-06
	W = 8 µm; L=0 µm	5.633803	1.934928	142	Fischer test	W = 2 µm; L=15 µm	< 2.2e-16
	2D	0	0	177			
5F	siCtrl - CK666	66	9.33381	50			
	siCtrl +CK666	39.39394	6.857605	33	Fischer test	siCtrl - CK666	0.05715
	siLmnA - CK666	75	10.40063	52			
	siLmnA + CK666	77.77778	11.59443	45	Fischer test	siLmnA - CK666	0.958
	siCtrl - blebbi	65.09434	4.629847	106			
	siCtrl + blebbi	54.73684	5.106819	95	Fischer test	siCtrl - blebbi	0.3309
	siLmnA - blebbi	79.41177	3.467229	136			
	siLmnA + blebbi	51.26051	4.582036	119	Fischer test	siLmnA - blebbi	5.44E-06
5G	siCtrl - CK666	19.7	2.507	33			
	siCtrl +CK666	27.46	5.988	13	Mann Whitney	siCtrl - CK666	0.5825
	siLmnA - CK666	25.56	3.196	39			

	siLmnA + CK666	22.69	3.627	35	Mann Whitney	siLmnA - CK666	0.095
	siCtrl - blebbi	15.62	1.456	69			
	siCtrl + blebbi	27.81	3.532	52	Mann Whitney	siCtrl - blebbi	< 0.0001
	siLmnA - blebbi	14.64	1.058	108			
	siLmnA + blebbi	23.42	2.218	61	Mann Whitney	siLmnA - blebbi	0.0004
5I	siCtrl - CK666	89.39394	15.56149	33			
	siLmnA - CK666	42.30769	6.774652	39	Fischer test	siCtrl - CK666	0.000161
	siCtrl +CK666	30.76923	8.533849	13	Fischer test	siCtrl - CK666	0.000773
	siLmnA + CK666	10	1.690308	35	Fischer test	siLmnA - CK666	0.004933
6A	Sun2WT	69.41177	4.997862	85			
	Sun2KO	67.90124	5.18729	81	Fisher test	Sun2WT	0.9885
	Sun2WT+siSun1	64.28571	4.840221	98	Fisher test	Sun2WT	0.7786
	Sun2KO+siSun1	57.5	5.526923	80	Fisher test	Sun2WT	0.2882
	Sun2KO+siSun1				Fisher test	Sun2WT+siSun1	0.6591
6E	% actin accumulation2.5 µm	9.090909	1.582524	33	Fischer test	% actin accumulation3 µm	9.55E-11
	% actin accumulation3 µm	83.72093	12.76732	43			
	% actin accumulation3.5 µm	28.26087	4.166838	46	Fischer test	% actin accumulation3 µm	5.66E-07
	% cell passage 2.5 µm	39.39394	6.857605	33	Fischer test	% cell passage 3 µm	0.04925
	% cell passage 3 µm	68.42105	11.09936	38			
	% cell passage 3.5 µm	90	16.43168	30	Fischer test	% cell passage 3 µm	0.1027

	% bead passage 2.5 µm	0	0	33	Fischer test	% bead passage 3 µm	9.33E-11
	% bead passage 3 µm	69.76744	10.63943	43			
	% bead passage 3.5 µm	97.4359	15.60223	39	Fischer test	% bead passage 3 µm	0.002486
	% nµclear passage 2.5 µm	90.90909	15.82524	33	Fischer test	% nµclear passage 3 µm	0.8681
	% nµclear passage 3 µm	94.73684	15.36835	38			
	% nµclear passage 3.5 µm	100	17.96053	31	Fischer test	% nµclear passage 3 µm	0.6544
S1H	1 µm	24.6	3.28	48	Mann Whitney	4 µm	0.726
	1.5 µm	15.5	2.24	32	Mann Whitney	4 µm	0.4593
	2 µm	14.04	2.046	24	Mann Whitney	4 µm	0.1942
	3 µm	19.15	4.841	13	Mann Whitney	4 µm	0.6567
	4 µm	13.78	1.077	9			
	5 µm	14.4	3.97	5			
S2D	1 µm	2.5	0.197642	160	Fisher test	5 µm	< 2.2e-16
	1.5 µm	45.58823	5.528386	68	Fisher test	5 µm	1.52E-11
	2 µm	66.66666	9.3352	51	Fisher test	5 µm	1.42E-05
	3 µm	81.66666	10.54312	60	Fisher test	5 µm	0.007662
	4 µm	94.11765	11.41344	68	Fisher test	5 µm	0.5325
	5 µm	98.33334	12.69478	60	Fisher test		
S2E	1.5 µm	13.43	2.068	30	Mann Whitne	5 µm	0.0006

					y		
	2 µm	10.47	1.43	34	Mann Whitney	5 µm	0.0362
	3 µm	8.122	0.6386	49	Mann Whitney	5 µm	0.1337
	4 µm	6.5	0.3698	68	Mann Whitney	5 µm	0.5569
	5 µm	6.583	0.3552	60			
S2F	1 µm	36.98	2.226	156			
	1.5 µm	33.49	2.629	37			
	2 µm	29.24	3.203	17			
	3 µm	15.45	2.893	11			
	4 µm	6.5	1.443	4			
	5 µm	8		1			
S4C	MyoIIWT_1.5 µm	69.49152	10.85275	60			
	MyoIIKO_1.5 µm	59.64912	10.22974	57	Fisher test	MyoIIWT_1.5 µm	0.5027
	MyoIIWT_3 µm	91.83673	13.69021	49			
	MyoIIKO_3 µm	89.28571	12.62691	56	Fisher test	MyoIIWT_3 µm	0.9189
S4D	MyoIIWT_1.5 µm_Cell	24.39	2.547	41			
	MyoIIWT_1.5 µm_Nucleus	15.07	1.989	41			
	MyoIIKO_1.5 µm_Cell	47	5.081	34	Mann Whitney	MyoIIWT_1.5 µm_Cell	0.0001
	MyoIIKO_1.5 µm_Nucleus	35.15	4.761	34			
	MyoIIWT_3 µm_Cell	11.16	1.136	45			
	MyoIIWT_3 µm_Nucleus	5.356	0.6921	45			
	MyoIIKO_1.5 µm_Cell	13.2	1.365	50	Mann Whitney	MyoIIWT_3 µm_Cell	0.0637

	MyoIIKO_1.5 µm_Nucleus	6.2	0.5707	50			
S5B	siCtrl_1.5µm	48.07692	6.928622	52			
	siArpC4_1.5µm	24.19355	5.438849	62	Fisher test	siCtrl_1.5µm	0.03119
	siCtrl_2µm	62.90323	6.134917	62			
	siArpC4_2µm	29.16667	5.356684	72	Fisher test	siCtrl_2µm	0.000467
	siCtrl_3µm	88.88889	3.959426	63			
	siArpC4_3µm	75.38462	5.343035	65	Fisher test	siCtrl_3µm	0.1447
	siCtrl_4µm	80.70175	5.227126	57			
	siArpC4_4µm	82.6087	4.563041	69	Fisher test	siCtrl_4µm	0.9641
S5C	siCtrl_1.5µm_Cell	30.56	3.391	25			
	siArpC4_1.5µm_Cell	33.13	5.767	15	Mann Whitney	siCtrl_1.5µm_Cell	0.8412
	siCtrl_1.5µm_Nucleus	20.12	2.53	25	Mann Whitney	siCtrl_1.5µm_Cell	0.005
	siArpC4_1.5µm_Nucleus	18.4	2.961	15	Mann Whitney	siArpC4_1.5µm_Cell	0.0202
	siArpC4_1.5µm_Nucleus				Mann Whitney	siCtrl_1.5µm_Nucleus	0.7035
	siCtrl_2µm_Cell	32.08	3.016	39			
	siArpC4_2µm_Cell	27.71	5.581	21	Mann Whitney	siCtrl_2µm_Cell	0.098
	siCtrl_2µm_Nucleus	21.31	2.483	39	Mann Whitney	siCtrl_2µm_Cell	0.0006
	siArpC4_2µm_Nucleus	19.48	5.356	21	Mann Whitney	siArpC4_2µm_Cell	0.0093

	siArpC4_2µm_Nucleus				Mann Whitney	siCtrl_2µm_Nucleus	0.0615
	siCtrl_3µm_Cell	19.11	1.296	56			
	siArpC4_3µm_Cell	12.15	1.226	49	Mann Whitney	siCtrl_3µm_Cell	< 0.0001
	siCtrl_3µm_Nucleus	10.38	0.8377	56	Mann Whitney	siCtrl_3µm_Cell	< 0.0001
	siArpC4_3µm_Nucleus	5.204	0.4061	49	Mann Whitney	siArpC4_3µm_Cell	< 0.0001
	siArpC4_3µm_Nucleus				Mann Whitney	siCtrl_3µm_Nucleus	< 0.0001
	siCtrl_4µm_Cell	15.04	1.034	46			
	siArpC4_4µm_Cell	12.98	1.258	57	Mann Whitney	siCtrl_4µm_Cell	0.0061
	siCtrl_4µm_Nucleus	8.783	0.7169	46	Mann Whitney	siCtrl_4µm_Cell	< 0.0001
	siArpC4_4µm_Nucleus	6.912	0.939	57	Mann Whitney	siArpC4_4µm_Cell	< 0.0001
	siArpC4_4µm_Nucleus				Mann Whitney	siCtrl_4µm_Nucleus	0.0004
S12B	siCtrl_1.5µm_Cell	17.64	3.04	36			
	siLmna_1.5µm_Cell	10.78	1.449	41	Mann Whitney	siCtrl_1.5µm_Cell	0.0043
	siCtrl_1.5µm_Nucleus	12.64	2.898	36	Mann Whitney	siCtrl_1.5µm_Cell	0.0043
	siLmna_1.5µm_Nucleus	6.024	1.167	41	Mann Whitney	siLmna_1.5µm_Cell	< 0.0001
	siLmna_1.5µm_Nucleus				Mann Whitney	siCtrl_1.5µm_Nucleus	0.0035
	siLmna_1.5µm_Nucleus				Mann Whitney	siLmna_2µm_Nucleus	0.3871

	siCtrl_2µm_Cell	8.917	0.9601	48			
	siLmna_2µm_Cell	8.488	0.754	41	Mann Whitney	siCtrl_2µm_Cell	0.8514
	siCtrl_2µm_Nucleus	4.792	0.7108	48	Mann Whitney	siCtrl_2µm_Cell	< 0.0001
	siLmna_2µm_Nucleus	4.707	0.5929	41	Mann Whitney	siLmna_2µm_Cell	< 0.0001
	siLmna_2µm_Nucleus				Mann Whitney	siCtrl_2µm_Nucleus	0.618
	siCtrl_3µm_Cell	4.959	0.3928	49			
	siLmna_3µm_Cell	5.537	0.466	41	Mann Whitney	siCtrl_3µm_Cell	0.145
	siCtrl_3µm_Nucleus	1.653	0.133	49	Mann Whitney	siCtrl_3µm_Cell	< 0.0001
	siLmna_3µm_Nucleus	1.915	0.1928	41	Mann Whitney	siLmna_3µm_Cell	< 0.0001
	siLmna_3µm_Nucleus				Mann Whitney	siCtrl_3µm_Nucleus	0.2402
	siLmna_3µm_Nucleus				Mann Whitney	siLmna_2µm_Nucleus	< 0.0001
S12C	siCtrl_1µm	5.769231	3.233357	52	Fisher test	siCtrl_3µm	< 2.2e-16
	siLmna_1µm	14	4.907138	50	Fisher test	siLmna_3µm	1.57E-11
	siLmna_1µm				Fisher test	siCtrl_1µm	0.3957
	siCtrl_1.5µm	67.92453	6.411533	53	Fisher test	siCtrl_3µm	0.02725
	siLmna_1.5µm	78.84615	5.663478	52	Fisher test	siLmna_3µm	0.9366
	siLmna_1.5µm				Fisher test	siCtrl_1.5µm	0.4554
	siCtrl_2µm	78.68852	5.243214	61	Fisher test	siCtrl_3µm	0.3123
	siLmna_2µm	75.92593	5.81799	54	Fisher	siLmna_3µm	0.7469

					test		
	siLmna_2µm				Fisher test	siCtrl_2µm	0.946
	siCtrl_3µm	89.09091	4.20368	55			
	siLmna_3µm	82	5.433231	50	Fisher test	siCtrl_3µm	0.6166
S13C	Sun2WT	38.62	5.618	26			
	Sun2KO	35.69	5.996	26			
	Sun2WT+siSun1	21.2	4.616	35	Mann Whitney	Sun2WT	0.0008
	Sun2KO+sisun1	23.48	5.216	33	Mann Whitney	Sun2KO	0.0757
S13D	Sun2WT	24.42	2.067	59			
	Sun2KO	17.93	1.744	55	Mann Whitney	Sun2WT	0.0011
	Sun2WT+siSun1	15.86	1.171	63	Mann Whitney	Sun2WT	< 0.0001
	Sun2KO+sisun1	21.62	2.63	45	Mann Whitney	Sun2WT	0.0743
	Sun2KO+sisun1				Mann Whitney	Sun2KO	0.332
S13E	Sun2WT_siCtrl	15.86	1.904	59			
	Sun2KO_siCtrl	10.62	1.082	55	Mann Whitney	Sun2WT_siCtrl	0.0115
	Sun2WT_siSun1	9.73	0.8912	63	Mann Whitney	Sun2WT_siCtrl	0.0016
	Sun2KO_siSun1	11.41	1.677	46	Mann Whitney	Sun2WT_siCtrl	0.0244
S14B	1 µm	62	6.86	50	Fischer test	2 µm	0.0006424
	1.5 µm	81.81	5.2	55	Fische	2 µm	0.2629

					r test		
	2 µm	92.59	3.56	54			
S14C	1 µm-cell	18.42	2.341	31		2 µm-cell	< 0.0001
	1 µm-Nucleus	14.84	2.229	31	Mann whitney	1 µm-cell	0.1525
	1.5 µm-cell	6.044	0.7939	45		2 µm-cell	0.0844
	1.5 µm-Nucleus	3.791	0.7103	43	Mann whitney	1.5 µm-cell	< 0.0001
	2 µm-cell	4.748	0.5854	50			
	2 µm-Nucleus	4.778	0.7630	27	Mann whitney	2 µm-cell	0.9783
S14D	Ctrl	62.	6.86	50			
	Y27632	19.607840	5.559	51	Fisher test	Ctrl	6.713E-05
	CK666	77.272730	6.317	44	Fisher test	Ctrl	0.271
S14E	Ctrl	18.42	2.341	31			
	Y27632	31.90	4.500	10	Mann whitney	Ctrl	0.0107
	CK666	11.44	1.225	34	Mann whitney	Ctrl	0.0202
S14G	1 µm	19.60784	5.559512	10	Fischer test	2 µm	7.139e-09
	1.5 µm	75.40984	5.51353	61	Fischer test	2 µm	0.717
	2 µm	82.5	6.007807	40			
S14H	1 µm-cell	31.90	4.500	10		2 µm-cell	< 0.0001

	1 µm-Nucleus	25.90	4.510	10	Mann whitne y	1 µm-cell	0.2896
	1.5 µm-cell	10.78	0.6852	46		2 µm-cell	0.3705
	1.5 µm-Nucleus	7.043	0.6278	46	Mann whitne y	1.5 µm-cell	< 0.0001
	2 µm-cell	9.788	0.7054	33			
	2 µm-Nucleus	6.000	0.7042	32	Mann whitne y	2 µm-cell	0.0001

Supplementary Discussion

Amoeboid versus mesenchymal migration

A recent article described a role for Myosin II based contraction at the front of the cell producing a pulling force on the nucleus[1]. Our results on Myosin II and actin localization as well as the results on Myosin II depletion and RICM imaging together rule out this mechanism for DCs migrating through constrictions. DCs are typical amoeboid cells, which move at high speed (mean velocity of 4.5 µm.min^{-1}), while HFFs cells studied in Petrie et al are slow mesenchymal cells (mean velocity of 10 µm.h^{-1},[1]). DCs move based on rear contraction and actin retrograde flow[2], they lack focal adhesions and do not rely on protrusive activity at the cell front. This is confirmed by the fact that Arp2/3 depletion makes these cells faster, while it reduces actin at the cell front, consistent with other recent studies on leukocytes[3,4]. We also recently showed that accumulation of Myosin II at the front of DCs, while required, together with Arp2/3 activity, for macropinocytosis, impedes cell migration[5]. Conversely, mesenchymal migration relies on actin polymerisation in front protrusions and on focal adhesions, with a front localisation of Myosin II, likely explaining the difference of mechanism for moving their nucleus through dense environments.

Actin structures and the cell nucleus

Several studies have reported the accumulation of actin filaments around the nucleus. A first set of articles have reported actin fibers resembling stress fibers (Actin cap[6] and TAN Lines[7]), but present on top of the nucleus and participating in its deformation, in mesenchymal cells plated on flat substrates [6–11]. A recent article also reported a formin (INF2) based nucleation of actin filaments around the nucleus triggered by external deformation imposed upon the cell and thus the nucleus[12]. While these phenomena might contribute to the formation of the actin accumulation we report for DCs migrating through constrictions, previously described perinuclear actin structures have a number of characteristics that make them likely to be different structures, with different functions, more related to mesenchymal cells. First, both the actin cap and TAN lines contain Myosin II and are associated to focal adhesions, forming arcs above the nucleus and deform it by exerting contractile forces[10]. They were also reported to interact with the nucleus through the LINC complex [6,7], and proposed to originate from focal adhesions thus being most likely nucleated by formins rather than Arp2/3. In complete contrast, the actin accumulation we report is based on Wave2 (Hem1)/Arp2/3 nucleator; we did not observe any Myosin II around the nucleus; the LINC complex is not required for the transmission of forces to the nucleus by the actin structure we observed, and the forces exerted on the nucleus seem to rather be compressive forces than contractile forces. Finally, we could not observe well defined contractile fibers and DCs do not require integrin based adhesion (and thus focal adhesions) to migrate[13,14]. Overall, these observations suggest that the actin structures we observed, while also associated to the nucleus, are different from structures previously reported in mesenchymal cells. Consistent with the observation of actin accumulation occurring also around confined passive objects (beads), we propose that the actin structures we observed result from confinement of a branched actin network, resulting in increased density of branches, leading to a strong pushing force, as previously proposed based on *in vitro* experiments[15].

Effect of LINC depletion on passage time through constrictions

A surprising result from our study is that disruption of the LINC complex decreases the cell and nuclear passage time through 2 µm wide constriction (Supplementary Fig. 13D, E) suggesting a negative role of the nucleo-cytoskeleton connection on the speed of cell passage. We also observed, consistent with the literature[16], that depletion of SUN proteins decreases cell persistence (Supplementary Fig. 13C), but not to an extent that could affect cell passage through constrictions. A recent study proposed that disconnecting the chromatin from the nuclear envelope would facilitate chromatin flow[17]. The LINC participates in the physical connection between the chromatin, the nuclear envelop and the cytoskeleton. It could thus reduce the capacity of chromatin to flow relative to these structures. Its depletion might thus facilitate the movement of chromatin during nuclear deformation through the constriction, which would be consistent with our results.

Supplementary References

1. Petrie, R. J., Koo, H. & Yamada, K. M. Generation of compartmentalized pressure by a nuclear piston governs cell motility in a 3D matrix. *Science* **345,** 1062–1065 (2014).
2. Liu, Y.-J. *et al.* Confinement and Low Adhesion Induce Fast Amoeboid Migration of Slow Mesenchymal Cells. *Cell* **160,** 659–672 (2015).
3. Wilson, K. *et al.* Mechanisms of leading edge protrusion in interstitial migration. *Nat. Commun.* **4,** (2013).
4. Pablo Vargas *et al.* Innate control of actin nucleation determines two distinct migration behaviours in dendritic cells. *Nat. Cell Biol.* (In Press).
5. Chabaud, M. *et al.* Cell migration and antigen capture are antagonistic processes coupled by myosin II in dendritic cells. *Nat. Commun.* **6,** 7526 (2015).

6. Khatau, S. B. *et al.* A perinuclear actin cap regulates nuclear shape. *Proc. Natl. Acad. Sci.* **106,** 19017–19022 (2009).
7. Luxton, G. W. G., Gomes, E. R., Folker, E. S., Vintinner, E. & Gundersen, G. G. Linear Arrays of Nuclear Envelope Proteins Harness Retrograde Actin Flow for Nuclear Movement. *Science* **329,** 956–959 (2010).
8. Gomes, E. R., Jani, S. & Gundersen, G. G. Nuclear Movement Regulated by Cdc42, MRCK, Myosin, and Actin Flow Establishes MTOC Polarization in Migrating Cells. *Cell* **121,** 451–463 (2005).
9. Versaevel, M., Grevesse, T. & Gabriele, S. Spatial coordination between cell and nuclear shape within micropatterned endothelial cells. *Nat. Commun.* **3,** 671 (2012).
10. Kim, D.-H., Chambliss, A. B. & Wirtz, D. The multi-faceted role of the actin cap in cellular mechanosensation and mechanotransduction. *Soft Matter* **9,** 5516–5523 (2013).
11. Gay, O. *et al.* RefilinB (FAM101B) targets filamin A to organize perinuclear actin networks and regulates nuclear shape. *Proc. Natl. Acad. Sci. U. S. A.* **108,** 11464–11469 (2011).
12. Shao, X., Li, Q., Mogilner, A., Bershadsky, A. D. & Shivashankar, G. V. Mechanical stimulation induces formin-dependent assembly of a perinuclear actin rim. *Proc. Natl. Acad. Sci. U. S. A.* **112,** E2595–2601 (2015).
13. Lämmermann, T. *et al.* Rapid leukocyte migration by integrin-independent flowing and squeezing. *Nature* **453,** 51–55 (2008).
14. Renkawitz, J. *et al.* Adaptive force transmission in amoeboid cell migration. *Nat. Cell Biol.* **11,** 1438–1443 (2009).

15. Parekh, S. H., Chaudhuri, O., Theriot, J. A. & Fletcher, D. A. Loading history determines the velocity of actin-network growth. *Nat. Cell Biol.* **7,** 1219–1223 (2005).

16. Lombardi, M. L. *et al.* The Interaction between Nesprins and Sun Proteins at the Nuclear Envelope Is Critical for Force Transmission between the Nucleus and Cytoskeleton. *J. Biol. Chem.* **286,** 26743–26753 (2011).

17. Schreiner, S. M., Koo, P. K., Zhao, Y., Mochrie, S. G. J. & King, M. C. The tethering of chromatin to the nuclear envelope supports nuclear mechanics. *Nat. Commun.* **6,** (2015).
